# Fate of Trace Organic Contaminants in the Hyporheic Zone: A Laboratory-Scale Column Experiment

**DOI:** 10.1021/acs.est.5c18582

**Published:** 2026-06-16

**Authors:** Edinsson Muñoz-Vega, Selina Hillmann, Mohammad Sajjad Abdighahroudi, Kai Ihle, Carolin Bertold, Christoph Schüth, Holger V. Lutze, Stephan Schulz

**Affiliations:** † Institute of Applied Geosciences, Technical University of Darmstadt, Darmstadt 64287, Germany; ‡ Hessian Agency for Nature Conservation, Environment and Geology (HLNUG), Groundwater, Wiesbaden 65203, Germany; § Chair of Environmental Analytics and Pollutants, Institute IWAR, Technical University of Darmstadt, Darmstadt 64287, Germany; ∥ Department Evolutionary Ecology & Environmental Toxicology, Goethe University Frankfurt, Frankfurt am Main 60438, Germany; ⊥ Water Resources Management Division, IWW Water Centre, Mülheim an der Ruhr 45476, Germany; # Centre for Water and Environmental Research (ZWU), Essen 45141, Germany; ¶ Kompetenzzentrum Wasser Hessen, Frankfurt Am Main 60438, Germany

**Keywords:** sorption, biotransformation, sediment organic matter, TrOCs mobilization

## Abstract

Rivers impacted by anthropogenic activities often contain numerous trace organic contaminants (TrOCs) that can infiltrate aquifers. The hyporheic zone, which is the interface between rivers and groundwater, can act as a natural attenuation barrier via sorption and biotransformation. To differentiate between these two processes, we conducted long-term laboratory column experiments using riverbed sediments from a creek downstream of wastewater treatment plants to quantify the fate of several ubiquitous TrOCs with diverse physicochemical properties. Triplicate columns were fed with either natural contaminated river water or with TrOC-free tap water to distinguish between attenuation mechanisms and to investigate potential remobilization. Mass balances and comparisons between experiments revealed a variety of attenuation behaviors. Certain hydrophobic (e.g., carbamazepine and tolyltriazole) or cationic compounds (e.g., metoprolol and sitagliptin) were retained by sediment sorption, whereas several polar anionic TrOCs remained largely mobile (e.g., hydrochlorothiazide and gabapentin), with some of them being biotransformed under prevalent reducing conditions (e.g., iopromide and oxipurinol). Overall, 10 out of 19 continuously detected TrOCs exhibited less than 25% removal over almost 300 pore volumes, reflecting limited biotransformation and the relevance of reversible sorption. These results suggest that while polluted hyporheic sediments of high organic matter content can temporarily reduce contaminant transport, their attenuation capacity is limited. Furthermore, previously sorbed pollutants may remobilize, posing long-term risks to the groundwater quality.

## Graphical abstract

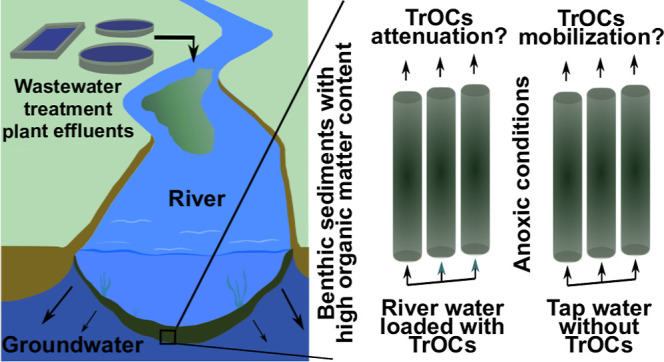

## 1 Introduction

Aquatic environments worldwide are increasingly impacted by trace organic contaminants (TrOCs) as a result of wastewater treatment plant (WWTP) effluent discharges and other human and industrial activities. This represents a threat to not only surface and groundwater bodies but also to sediments, soils, and linked ecosystems.^1–3^ Understanding the processes controlling the transport and attenuation of TrOCs in aquatic systems is therefore essential for assessing their environmental impact and for developing effective mitigation strategies.^4^

TrOCs comprise a highly diverse group of substances, including pharmaceuticals, personal care products, and industrial chemicals, that differ widely in their physicochemical properties and environmental behavior. This diversity contributes to the complexity of predicting their fate and transport.^5,6^ Recent studies with sites from many countries have demonstrated their widespread occurrence in rivers, highlighting their global relevance and the need for a better understanding of their environmental dynamics.^7^

Within river systems, the hyporheic zone (HZ), which is the interface between streams and groundwater,^8^ is often regarded as a critical compartment for TrOC attenuation, as it constitutes a natural barrier against contamination of adjacent groundwater resources that are essential for drinking water production.^9,10^ This functionality results mainly from sorption onto sedimentary organic matter, mineral phases, and biofilms, as well as biotransformation mediated by the diverse microbial communities present in these environments.^11^ These two processes are inherently complex because they are governed by the physicochemical properties of the TrOCs (e.g., speciation and polarity), by the hydrogeochemical and hydraulic conditions of the sediment–water matrix (e.g., pH, redox potential, and residence time),^12^ and by factors such as temperature and microbial community dynamics.^13^ Consequently, distinguishing between these two attenuation pathways is often challenging because of the highly variable hydrogeochemical conditions that characterize natural HZ environments.^14^

One approach is to consider both processes together in terms of the “relative attenuation” of TrOCs, quantified by comparing compound concentrations in pore water of the riverbed with those in the surface water,^15,16^ not allowing for the differentiation between the two processes. Other approaches aiming to characterize both mechanisms separately have combined numerical modeling of field experiments across different temporal and spatial scales^17,18^ as well as laboratory-based determination of sorption and biotransformation parameters.^19,20^ The estimation of sorption coefficients and, in particular, first-order biotransformation rates has been a valuable technique to understand TrOCs’ fate but is subject to considerable uncertainty.^21^ Such estimates, especially under field conditions that are highly transient and characterized by a high degree of heterogeneity,^19^ typically depend on simplified assumptions, such as (i) steady-state conditions and invariant pore water velocities,^22^ (ii) spatially homogeneous microbial activity within the reactive domain represented by constant removal rates over time and space,^18^ (iii) instantaneous equilibrium sorption,^19,20^ (iv) the absence of mass transfer between mobile and immobile water regions,^23^ (v) simplified representations of the hyporheic exchange mechanisms,^24^ and (vi) uncertainties resulting from discontinuous observations of TrOC concentrations.^25^ Consequently, it remains unclear to what extent sorption and biotransformation can be reliably distinguished, and how compound-specific properties contribute to these processes in complex water–sediment systems, such as the HZ.

Therefore, to analyze the attenuation of TrOCs in the HZ, we conducted a series of laboratory column experiments with riverbed sediment cores from a creek downstream of wastewater treatment plants in the Hessian Ried, Germany. We used polluted river water as well as TrOC-free tap water as feedwater to evaluate the relative contributions of biotransformation and sorption and to assess TrOC mobilization from the contaminated benthic sediments, an aspect that remains poorly explored in HZ studies. Periodic sampling over approximately 300 pore volumes allowed the quantification of the relative attenuation (RA) of 19 TrOCs that are consistently present in the stream and were carefully selected to span a broad range of physicochemical properties expected to influence their environmental fates. By comparing the results obtained from the different experimental setups, we aim to quantify and distinguish between the biotransformation and sorption processes. With this approach, we intend to improve the characterization of TrOCs’ attenuation and its underlying mechanisms within the HZ.

## 2 Methods

### 2.1 Riverbed Cores Collection and Experimental Setup

The sediments used in this study were collected from the riverbed of the Landgraben, a lowland stream near the city of Trebur, in the Hessian Ried, Germany (coordinates: 49°55′13.8″N, 8°23′55.2″E; Figure S1). The Landgraben has been receiving effluents from several WWTPs along its course for decades,^26,27^ resulting in a continuous presence of TrOCs^28^ along the stream and measurable occurrence on adjacent groundwater (Tables S1 and S2).

In total, 11 riverbed sediment cores were collected on October 28, 2024 using a soil core sampler with a liner of 30 cm length and 5 cm diameter (Eijkelkamp, The Netherlands). The cores were collected randomly within an area of approximately 30 m^2^, spanning 6 m along a transect perpendicular to the flow direction and 5 m parallel to the flow path. A minimum distance of 1 m from the riverbanks was maintained to target more homogeneous microhabitats. After collection, the cores were immediately capped at both ends and transported vertically to preserve the internal structure. No visible structural disturbances (e.g., cracks or loss of stratification) were observed during the transport.

At the time of sampling, the river water temperature was 13.5 °C, which is close to the annual average of 13.8 ± 5.7 °C (15 September 2024 to 14 September 2025; Figure S2). The column experiments were conducted at a constant room temperature of 20 °C, which is higher than the in situ temperatures during the experimental period (October–March), but falls within the observed seasonal temperature range of the study site, particularly during late spring and summer months. Thus, the laboratory temperature remains within environmentally relevant conditions.

Hydrochemical data from nearby monitoring stations operated by the Hesse State Office for Nature Conservation, Environment, and Geology (HLNUG) indicate a redox-stratified system in the study area (Section S1). Surface water is characterized by oxic, nitrate-bearing conditions (mean O_2_: 0.26 mM; mean NO_3_^–^: 0.11 mM), whereas groundwater exhibits strongly depleted O_2_ and NO_3_^–^ concentrations (Table S4). These data indicate the presence of reducing to anoxic conditions in the subsurface sediments of the study area. Sulfate is present at comparable concentrations in both surface water and groundwater (mean river: 1.02 mM; mean groundwater: 1.18 mM), indicating a substantial background concentration. Furthermore, recent redox data collected directly in the riverbed of the Landgraben stream indicate anoxic and reducing conditions at depths of 5 cm and below (Figure S4).

Of the 11 collected cores, six were used for column experiments, and the remaining five were divided into three equal subsections each for sediment characterization. Physicochemical properties of the sediments are presented in Table 1, and the major elemental composition is in Table S5.

**Table 1:** Physicochemical Properties of Riverbed Sediments^a^

Parameter	Depth 0–10 cm	Depth 10–20 cm	Depth 20–30 cm
mean particle size [mm]	0.15 ± 0.00	0.17 ± 0.03	0.13 ± 0.04
organic matter content [%]^b^	3.9 ± 0.7	7.8 ± 2.1	5.9 ± 2.1
organic carbon content [%]^c^	1.1 ± 0.2	2.5 ± 1.0	1.8 ± 0.7
CEC [cmol+/kg]^d^	5.9 ± 1.1	10.1 ± 3.3	8.7 ± 3.6

a Values are reported as averages with standard deviations from five samples. Individual measurements are provided in Table S6.

b Loss on ignition.

c Total organic carbon analyzer.

d Cation exchange capacity determined according to ISO 11260:2018.^29^

The experimental setup consisted of six acrylic glass columns (5 cm diameter, 25 cm length) each filled with one of the riverbed cores. The cores used for the column experiments were installed immediately after transport. The original vertical orientation of the cores was preserved, with the upper sediment layer positioned at the inflow of the columns. For installation, the liners were rotated 180° and carefully cut, and the cores were gently transferred into the acrylic columns, which were prefilled with the respective feed solution (see below) to prevent desaturation and limit air entrapment. After installation, the columns were capped and left overnight to allow gravitational settling prior to flow initiation. Two sets of triplicate experiments were conducted differing in the feeding solution, as detailed below and presented in Figure 1:

1) River water-fed columns (CR1, CR2, and CR3): Three columns were fed with river water collected every 2 weeks from the same location where the soil cores were extracted. For each sampling event, 100 L of river water was collected and distributed into four 25 L glass bottles, which were stored refrigerated at 4 °C without headspace, and kept sealed. The collected water was used over a period of approximately 14 days, with individual bottles supplying the columns for 3 to 4 days each. Each bottle was removed from refrigeration approximately half day prior to use to allow temperature equilibration. The storage tank supplying the columns was replenished every 3 to 4 days, to ensure regular renewal of the river water supplied to the columns throughout the experiment. This set of triplicates was designed to characterize the transport behavior of TrOCs in the HZ mimicking natural field conditions.
2) Tap water-fed columns (CT1, CT2, and CT3): To further assess reactive transport characteristics as well as potential TrOC mobilization from the contaminated sediments of the riverbed, the second set of triplicates was fed with tap water free of TrOCs, allowing the evaluation of desorption processes from the sediment matrix. Tap water was selected as a readily available, TrOC-free baseline for assessing the sediment-derived contributions. This choice is supported by the fact that the river water at the study site consists largely of treated wastewater, much of which originates from tap water. In addition, its ionic strength (0.012 M) was similar to that of river water (0.018 M), ensuring comparable geochemical conditions. Tap water was collected at the same frequency as river water.

**Figure 1:**
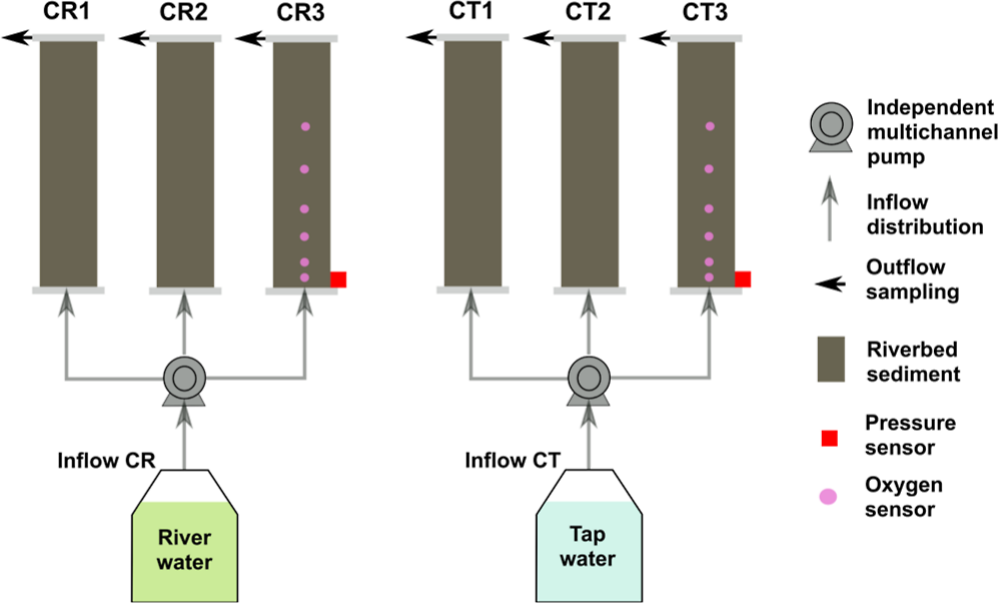
Scheme of the experimental setup. CR and CT represent columns fed with river water and tap water, respectively.

All columns were operated under saturated conditions in the upflow direction using two multichannel pumps (Ismatec, Switzerland) equipped with short PharMed tubing (2.54 mm diameter, Coleparmer, Germany) and connected to the bottoms of the columns by stainless steel tubing (1.52 mm diameter). During the full duration of the experiment, the columns were continuously operated with a flow rate of 0.53 L/d, resulting in a seepage velocity of 27 cm/d, similar to previous studies in the HZ of polluted streams.^22,30^ Porosity, determined based on measured dry bulk density (determined gravimetrically, ISO 11272) and particle density (helium pycnometer, AccuPyc, ISO 17892-3) of short metal cylinder samples (250 mL) directly inserted into the riverbed sediment, thereby minimizing disturbance during sampling (*n* = 10), yielded an average value of 55%. On the basis of this, the applied flow rate corresponds to approximately two pore volumes per day, with an equivalent residence time of 12.2 h. The experiment was conducted from October 2024 to March 2025, spanning 142 days, resulting in the exchange of 279 pore volumes in each column and a total of 11 different sampling events of feedwater from the stream.

For both sets of triplicates, one column was equipped with six oxygen spot sensors (Presens, Germany) to measure dissolved oxygen (DO) concentrations. Additionally, a pressure transducer (SICK, Germany) was installed in the inflow section of one column per triplicate to measure the evolution of hydraulic conductivity (Figure 1). Pressure data were recorded with a data logger (CR1000X, Campbell Scientific).

### 2.2 Columns Water Sampling and Analytical Chemistry

Inflow water samples were collected following each river water sampling event and tap water collection (*n* = 11), plus two additional samples at the beginning of the experiment, taken from the initially used water and from the renewed water on day 3, to ensure there were no significant changes in water composition during storage (Section S6). Outflow samples were collected at different time intervals. At the start of the experiment, sampling was performed at a higher frequency (nine sampling events within the first 16 days, including two samples per day during the first 2 days) to characterize the initial conditions of the riverbed cores and to capture the expected desorption dynamics. Subsequently, samples were consistently collected on days 2 and 14 after switching to a new batch of river water. This approach was intended to capture the behavior of the different river water compositions with a limited number of samples. In total, 28 outflow samples were taken from each of the columns.

The 194 (26 inflow and 168 outflow) water samples collected were analyzed for dissolved organic carbon (DOC) with a carbon analyzer (VarioTOC, Elementar, Germany), major ions with an ion chromatograph (Metrohm 882 Compact IC plus, Germany), and trace elements with an ICP-MS (Analytik Jena Plasma Quant MS, Germany). pH and electrical conductivity were measured with a portable multimeter (HACH, Germany), same as for DO of inflow samples.

All samples were analyzed for 22 TrOCs using an HPLC–MS/MS. The selection of target compounds was carefully designed to cover a wide range of physicochemical properties, particularly with respect to polarity (log *D*) and ionic speciation, two properties that strongly affect sorption dynamics^31,32^ and biotransformation,^6^ while also reflecting compounds consistently detected in the river water at the study site based on the previous monitoring data (Table S1). The physicochemical properties of the substances are provided in Table S7, and the description of the analytical method is provided in Section S4 of the Supporting Information.

### 2.3 Mass Balances and Relative Attenuation (RA) Calculation

Given the controlled experimental conditions with respect to water flow rate and duration, as well as the relatively large number of samples collected, mass balance calculations were performed by comparing the total injected mass with the total mass recovered in the outflow samples. Accordingly, we define the term Δ*M* as

1 $$\Delta M_{i}^{j}=\sum_{t=0}^{t=t_{f}}M_{out}{\left(t\right)}_{i}^{j}-\sum_{t=0}^{t=t_{f}}M_{in}{\left(t\right)}_{i}^{j}$$

where Δ*M*_*i*_^*j*^ represents the mass balance for chemical species *i* in column *j*. Terms *M*_out_(*t*)_*i*_^*j*^ and *M*_in_(*t*)_*i*_^*j*^ refer to the mass of species *i* in the outflow and inflow, respectively, at time *t* in column *j*. For TrOCs, we additionally define the RA factor, as the fraction of the injected mass that is not recovered in the outflow as follows:

2 $$RA_{i}^{j}=1-\frac{\sum_{t=0}^{t=t_{f}}M_{out}{\left(t\right)}_{i}^{j}}{\sum_{t=0}^{t=t_{f}}M_{in}{\left(t\right)}_{i}^{j}}$$

In eq 2, RA_*i*_^*j*^ represents the RA of compound *i* in column *j* for the columns fed with river water. The summation is performed over the entire experimental duration, from the start of the experiment (*t* = 0) to the final sampling time (*t* = *t*_f_), with the measured concentrations being linearly interpolated and multiplied by the volume of water injected.

RA can be negative when the cumulative outflow mass exceeds the cumulative inflow mass, which may occur for compounds undergoing significant desorption or production as transformation products of other substances. For compounds exhibiting conservative behavior, the cumulative outflow should closely match the cumulative inflow, i.e., RA ≈ 0. Conversely, for compounds with low mass detected in the outflow, indicating high attenuation, RA ≈ 1. With this definition, positive values indicate net attenuation, whereas negative values indicate net mobilization or production.

Since the tap water used in the desorption set of triplicates was free of TrOCs, we defined a relative mobilization (RM) factor for that group of columns:

3 $$RM_{i}^{k}=\frac{\sum_{t=0}^{t=t_{f}}M_{out}{\left(t\right)}_{i}^{k}}{\frac{1}{n}\sum_{j=1}^{n}\sum_{t=0}^{t=t_{f}}M_{out}{\left(t\right)}_{i}^{j}}$$

In eq 3, RM_*i*_^*k*^ represents the RM of compound *i* in column *k*. The normalization is made by the average cumulative outflow mass of the three river water-fed columns. This normalization allows for a direct comparison of the mass recovered from the tap water columns to the mass retrieved from the columns fed with river water. RM would be zero for compounds for which no desorption was observed in the tap water columns. RM = 1 would imply that the same amount of outflowing mass over 279 pore volumes in the river water-fed columns was mobilized from the tap water-fed ones. Thus, RM provides a measure of the extent to which the mass attenuation observed in the river water-fed columns is associated with reversible retention and subsequent desorption processes.

## 3 Results and Discussion

### 3.1 General Hydrogeochemical Conditions

The experiments were characterized by strong DO consumption within all columns, particularly in those fed by river water, where anoxic conditions developed in the first few millimeters and extended along the entire flow path (first measurement point at 2.5 cm, Figure 2), similar to field conditions (Figure S4). In the tap water columns, suboxic conditions were reached within the first 7 cm after approximately two months of operation and constantly anoxic conditions toward the column’s outlet (Figures 2 and S5). Simultaneously, complete denitrification occurred in all columns (Figures 2 and S6) over the full duration of the experiment. The strong reduction of these two electron acceptors was accompanied by slight DOC attenuation in the river water-fed columns and by DOC mobilization in the tap water columns (Figures 2 and S7). To better understand these observations, Table 2 presents the mass balances (eq 1) of the major species involved in DOC oxidation.^33,34^ Besides minor Mn and Fe reduction, a substantial production of SO_4_^2–^ occurred in both sets of triplicates (i.e., no sulfate reducing conditions), which we attribute to the presence of monosulfides and/or iron sulfides in the sediments, being oxidized by DO and NO_3_^–^. Furthermore, electron balance calculations show that, assuming DOC oxidation provides 4 e^–^ per mole of carbon, sulfide oxidation yields 7 or 8 e^–^ per mole,^33^ and, on the other hand, the reduction of DO and NO_3_^–^ requires 4 e^–^ and 5 e^–^, respectively, a significant deficit of electron donors remains. This deficit suggests additional oxidation of organic carbon from the relatively high organic matter content of the riverbed sediments (Table 1). Overall, our results indicate that reducing conditions prevailed in all columns, with anoxic conditions developing in large parts of the flow path, sustained by the high organic carbon content of the sediments. These results are consistent with previous field studies that report rapid oxygen depletion and the onset of denitrification within only a few centimeters below the surface water-sediment interface for lowland streams^9,15,18,24^ and are further supported by the reducing conditions observed in the adjacent groundwater.

**Figure 2:**
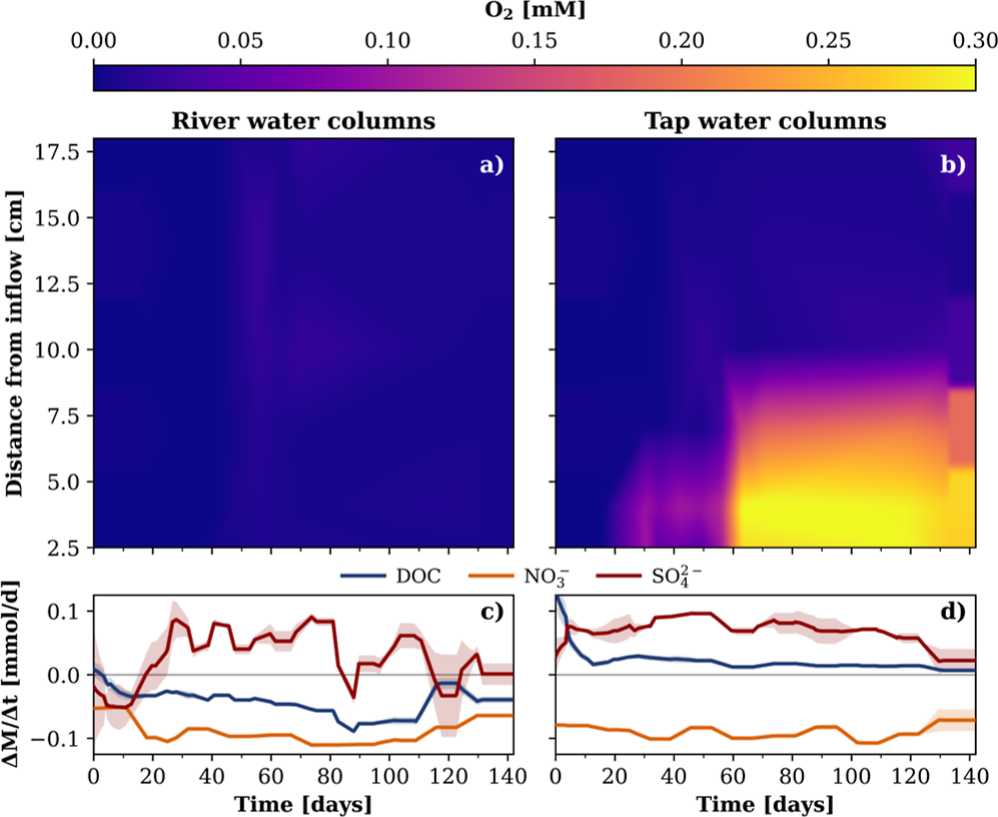
Temporal evolution of redox species in the column experiments. (a, b) DO (O_2_) concentration profiles in the river water (CR3) and tap water (CT3) columns, respectively. (c, d) Mean mass change rates (Δ*M*/Δ*t*) of DOC, NO_3_^–^, and SO_4_^2–^. Lines represent the average across triplicates, and shaded areas indicate the standard deviation.

**Table 2:** Mass Balances of DOC and Major Electron Acceptors^a^

species	Δ*M* CR [mmol]^b^	Δ*M* CT [mmol]^b^
DOC	–6.28 ± 0.13	2.67 ± 0.44
O_2_	–20.24 ± 0.00	–20.36 ± 0.00
NO_3_^–^	–12.83 ± 0.03	–12.65 ± 0.38
Mn	0.30 ± 0.08	0.55 ± 0.00
Fe	0.01 ± 0.00	0.03 ± 0.01
SO_4_^2–^	4.63 ± 1.25	9.32 ± 0.46

a Positive values of Δ*M* indicate net release, whereas negative values indicate net consumption. The temporal evolution of these species in all columns is provided in Section S5.

b Average and standard deviations of three columns.

### 3.2 TrOC Occurrence in River Water and Breakthrough Behavior in Column Experiments

Concentrations of the selected TrOCs in the river water samples are shown in Figure 3, sorted by their median values. Of the 22 analyzed compounds, 19 were consistently detected in the Landgraben stream. The only exceptions were ciprofloxacin, ritalinic acid, and atenolol, which were not quantified in any sample. Among the detected compounds, several exhibited pronounced variabilities in concentration over time, as indicated by the large interquartile ranges, reflecting the transient nature of TrOC mass fluxes in the river. Observed concentrations spanned from only a few ng/L, such as for fluconazole (FCZ; median = 55 ng/L), up to several μg/L, such as for iopromide (IOP; median = 2090 ng/L). Overall, the TrOCs identified in the Landgraben cover a broad spectrum of speciation and polarity (Table S7), at a pH value of 7.8, which corresponds to the mean pH measured during the column experiments (Figure S11).

**Figure 3:**
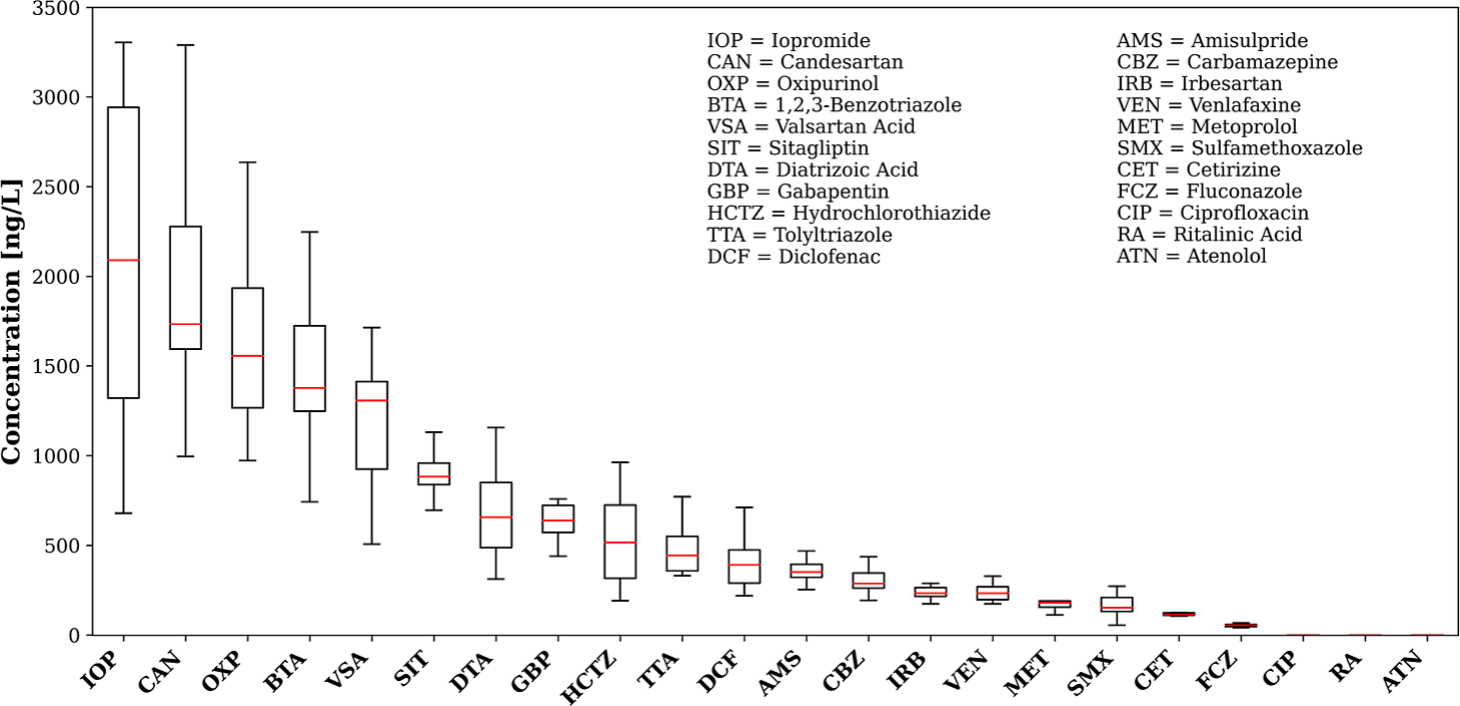
Boxplots of TrOC concentration in the Landgraben river over 11 sampling events conducted between 28 October 2024 and 04 March 2025. The red lines represent the median concentration of each compound.

Breakthrough curves (BCs) for all 19 compounds in the six columns are provided in Section S6, while Figure 4 shows the BCs of four representative compounds exhibiting clearly distinct behaviors. There, sitagliptin (SIT) displayed consistently lower outflow than inflow concentrations in the river water-fed columns, whereas it was continuously eluted from the CT columns throughout the entire experiment. Cetirizine (CET) and metoprolol (MET) showed similar behavior (Figures S12 and S13, respectively).

**Figure 4:**
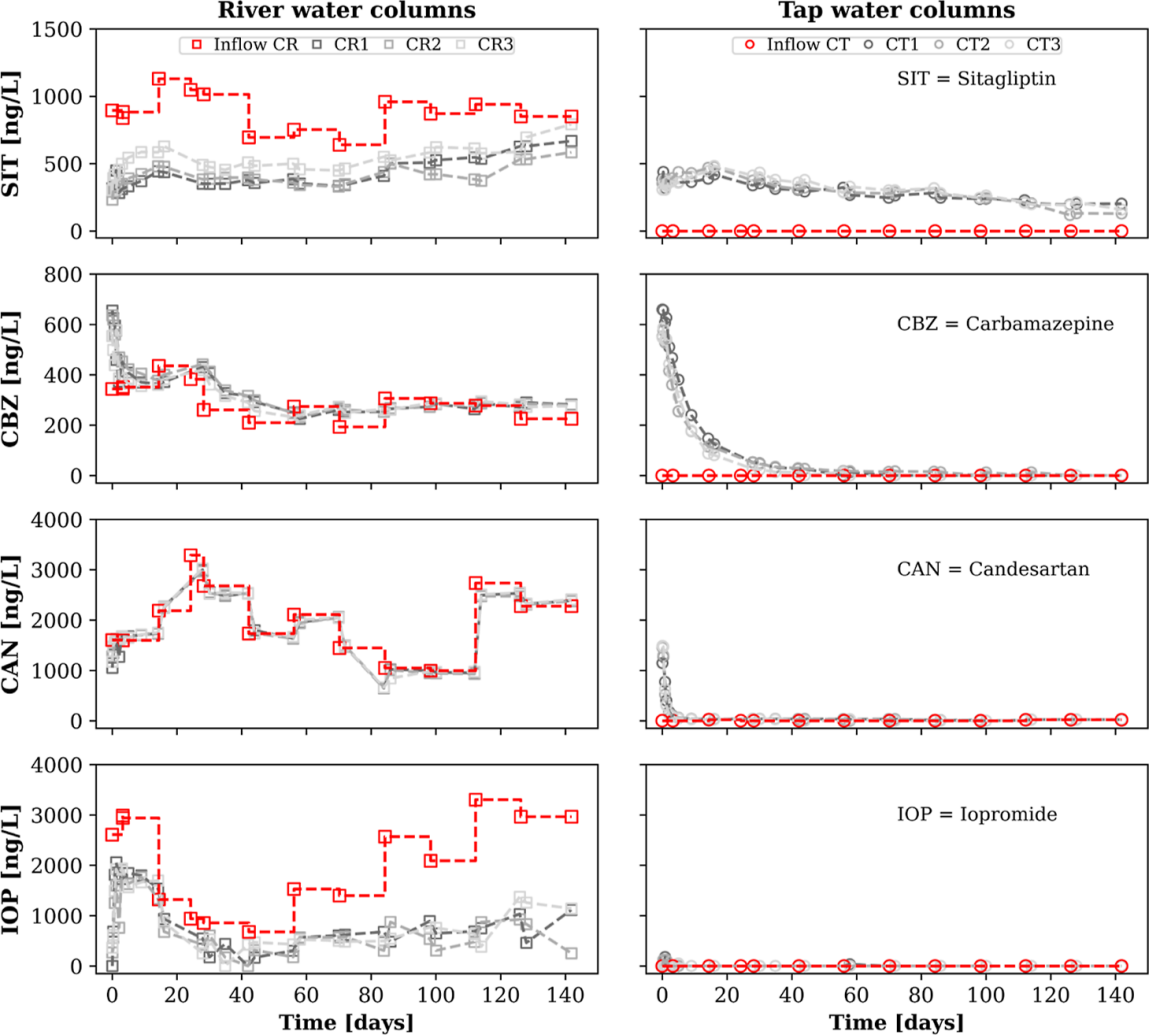
BCs of sitagliptin, carbamazepine, candesartan and iopromide in the six columns.

A second group of compounds is exemplified by carbamazepine (CBZ; Figure 4), which in the river water-fed columns exhibited outflow concentrations that generally followed the inflow patterns, albeit with minor deviations, and desorbed from the CT columns after approximately two months (roughly 120 pore volumes). This behavior is representative of several other compounds, including 1,2,3-benzotriazole (BTA), tolyltriazole (TTA), diclofenac (DCF), and irbesartan (IRB) (Figures S14, S15, S16, and S17, respectively).

A third class is represented by candesartan (CAN; Figure 4), which behaved mostly conservatively in the river water-fed columns, while in the CT columns, it exhibited a rapid leaching of resident pore water in the collected sediment cores. Fluconazole (FCZ) and hydrochlorothiazide (HCTZ) showed a similar pattern (Figures S18 and S19, respectively). Gabapentin (GBP) also followed this behavior but exhibited lower outflow concentrations than the corresponding inflows toward the end of the experiment (Figure S20).

Another clearly distinguishable group is represented by iopromide (IOP; Figure 4), which consistently displayed lower outflow than inflow concentrations in the CR columns and was almost absent in the CT outflows. This behavior was also observed for amisulpride (AMS), diatrizoic acid (DTA), oxipurinol (OXP), sulfamethoxazole (SMX), and venlafaxine (VEN) (Figures S21, S22, S23, S24, and S25, respectively).

Finally, valsartan acid (VSA) deviated from all other compounds by consistently showing higher outflow than inflow concentrations in the river water-fed experiments, along with a rapid elution of initial pore water from the CT columns (Figure S26).

Overall, the distinctly different behaviors observed across the studied TrOCs highlight the suitability of the selected compounds for capturing different attenuation processes in the HZ. Detailed explanations and interpretations of these dynamics are provided in the following sections.

### 3.3 RA and RM of TrOCs

On the basis of the measured BCs, RA (eq 2; Figure 5) and RM (eq 3; Figure 5) of the TrOCs were quantified throughout the entire experiment and interpreted regarding their polarity (log *D*) and speciation at the experimental pH. The investigated compounds exhibit a broad and continuous attenuation spectrum, ranging from negative values (VSA, TTA, and CBZ) to almost complete attenuation over the experimental duration of 279 pore volumes, as observed for AMS and VEN.

**Figure 5:**
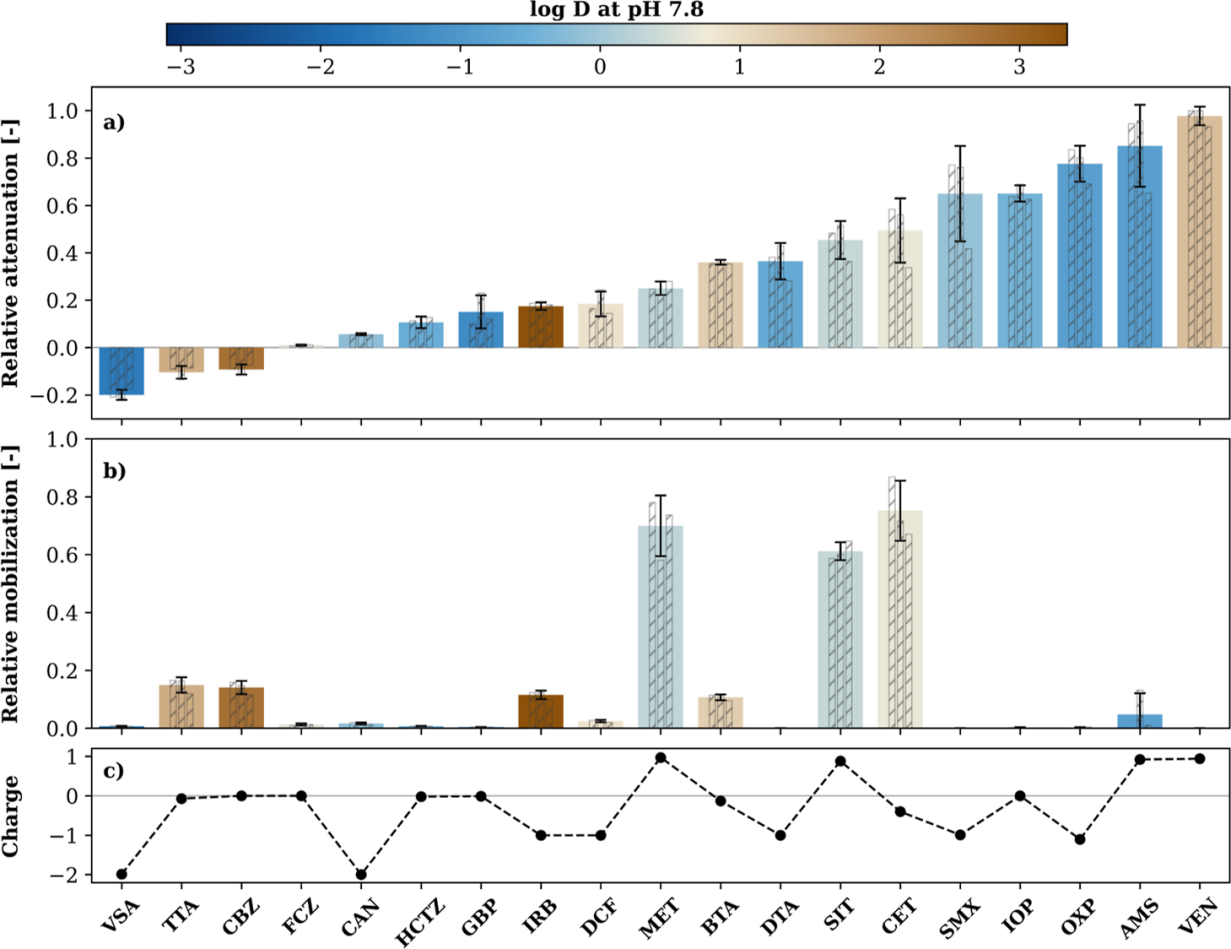
(a, b) Average RA and RM of the detected TrOCs for river and tap water-fed column experiments (*n* = 3), respectively. Bar colors represent the log *D* (blue tones = more hydrophilic; brown tones = more hydrophobic). Error bars indicate the standard deviation of the triplicate measurements, while dashed bars show individual replicate values. (c) Average molecular charge at the experimental pH.

#### 3.3.1 Negatively Attenuated Compounds

In the case of VSA, its RA was negative and its RM was almost zero (Figure 5). Since VSA is a strong anion and highly polar under the experimental conditions, it is indicated that this is not linked to desorption processes, as sorption on organic matter-rich sediments is generally not expected to be relevant for anionic compounds with low log *D*.^35^ A likely explanation is the presence of the parent compound valsartan, given its widespread consumption as an antihypertensive agent in Germany.^36^ Moreover, VSA can be a transformation product of other compounds from the sartan group,^20,37,38^ among which at least CAN and IRB were constantly detected in the river water (Figure 3). These observations suggest that, under the prevalent anoxic conditions in the river water-fed columns (Table 2), VSA was formed from parent compounds over relatively short time scales.

The negative attenuation observed for CBZ and TTA has a different explanation than that proposed for VSA. Both compounds are predominantly neutral under the experimental conditions and exhibit relatively high log *D* values (2.77 and 1.78, respectively), which indicate a greater affinity for sedimentary organic phases. In addition, CBZ^12,39^ and TTA^40^ are known to be poorly biotransformed in the environment. This, together with the fact that the RM of both compounds (Figure 5) closely matches their RA (Figure 5), indicates that both compounds are not attenuated in the analyzed river sediments but mobilized mainly at the beginning of the experiments through desorption.

#### 3.3.2 Compounds Dominated by Reversible Sorption

The relevance of sorption in the attenuation of TrOCs becomes more evident for compounds such as CET, MET, and SIT, for which a high RM was observed (Figure 5). The strong sorption of cationic compounds, such as MET and SIT, is expected on natural sediments at a pH value higher than neutral due to electrostatic interactions and cation exchange processes.^41^ The fact that the RM of MET is much higher than its attenuation, together with the observation that outflow concentrations occasionally reached inflow concentrations in the river water-fed columns (Figure S13), suggests that retention of MET is limited and readily reversible under the investigated conditions. In contrast to MET, SIT exhibits a higher attenuation but still substantial mobilization. Despite the increased attenuation relative to MET, mobilization remains dominant, indicating that retention of SIT is largely reversible. This behavior supports that biotransformation does not represent a major attenuation pathway for SIT, consistent with previous studies.^42,43^ Interestingly, CET exhibited the highest RM among all analyzed compounds (RM = 0.75). CET is a zwitterion of high lipophilicity at the experimental pH.^44^ Furthermore, CET has been reported to show no biotransformation under anoxic conditions;^45^ therefore, we attribute its attenuation mainly to reversible sorption, likely driven by hydrophobic interactions of its cationic fraction.

#### 3.3.3 Conservative and Weakly Attenuated Compounds

Among all analyzed TrOCs, FCZ showed the most conservative behavior, with RA ≈ 0 and only minimal RM, which we attribute to the initial resident pore water. This conservative behavior agrees with previous studies reporting the non-sorbing and highly persistent nature of FCZ,^11,46,47^ supporting its suitability as a tracer of WWTP effluents in the environment. In parallel, the RA ≈ 0 observed for FCZ in our experiment further validates the linear interpolation approach used to derive mass balances. CAN exhibited a similar pattern, showing persistent and mobile behavior consistent with previous findings.^48^ We attribute the behavior of both compounds to their preferential distribution into the aqueous phase due to their low log *D* values.

Other groups of compounds for which we observed a RM higher than that of FCZ and CAN were IRB, BTA, and DCF (Figure 5), suggesting that sorption is at least partially responsible for their attenuation. Among these three, IRB exhibited the lowest attenuation and the highest mobilization, which persisted for approximately 100 days in the CT columns (Figure S17). Despite its predominantly anionic speciation under the experimental conditions, IRB has the highest log *D* (3.34, Table S7) of all analyzed TrOCs, showing its hydrophobic nature and affinity for sorption onto sedimentary organic matter. IRB has been reported to transform into VSA,^20,38^ although it is more efficient under oxic conditions. In line with this, our results indicate that sorption was the dominant, although still mild, attenuation mechanism for this compound. In the case of DCF, which is also anionic but has a log *D* lower than that of IRB, the significantly higher RA compared to its RM suggests that biotransformation was the dominant removal pathway. DCF is known to undergo efficient biotransformation under denitrifying and manganese-reducing conditions,^49^ both of which prevailed in our experiments. BTA, which is mostly neutral under the experimental conditions and more hydrophobic than DCF, showed continuous desorption in the CT columns, again pointing to sorption as an important attenuation mechanism. Nevertheless, high RA during the elevated concentrations in the river water from day 80 until the end of the experiment (Figure S14) compared to a moderate RM suggests that biotransformation also occurred. This is in agreement with reports of BTA degradation under anoxic conditions.^18^ Interestingly, other studies have observed that BTA biodegradation under anoxic conditions often exhibits a pronounced lag phase of 14–60 days,^50,51^ similar to our observations. Although the sediments in our experiment had already been exposed to BTA prior to the experiment, the delayed onset of biotransformation might be related to the adaptation of the microbial community to altered laboratory conditions.

#### 3.3.4 Compounds Attenuated under Prevalent Anoxic Conditions

Of the remaining compounds not discussed so far, DTA, SMX, OXP, HCTZ, GBP, IOP, and VEN did not exhibit substantial mobilization in the CT columns (≤0.01 RM; Figure 5), initially suggesting that sorption was not a relevant attenuation mechanism for these compounds. For DTA, SMX, and OXP in particular, sorption is indeed highly unlikely because these three compounds were predominantly anionic and rather hydrophilic under the experimental conditions, with log *D* values below zero. The high RA of DTA, SMX, and OXP (Figure 5) therefore indicates biotransformation as the dominant process. Biotransformation of SMX^52,53^ and DTA^54,55^ under anaerobic conditions has also been previously reported. Notably, we are not aware of studies demonstrating the biotransformation of OXP, which has been characterized as recalcitrant in aerobic treatment systems^56^ and during sequential managed aquifer recharge,^48^ yet it appears that our experimental conditions (anoxic) allowed for substantial biotransformation (Figure S23).

HCTZ, GBP, and IOP are characterized by a neutral speciation, which in principle could favor sorption. However, they are also among the most hydrophilic compounds (log *D* < 0, Table S7), especially when compared to BTA, CBZ, CET, and TTA, which are more hydrophobic (log *D* > 0, Table S7) and for which we observed desorption from the CT columns. This agrees with previous studies highlighting the relevance of log *D* for the transport of neutral TrOCs.^35,57^ Therefore, the RA observed for HCTZ, GBP, and IOP most likely originates from transformation reactions rather than sorption. The low attenuation of HCTZ (RA ≈ 0.1) is consistent with earlier studies reporting limited biotransformation of this compound under different redox conditions.^20,58^ GBP exhibited a slightly higher but still mild removal (RA ≈ 0.15), which is also in line with studies describing its biotransformation under denitrifying conditions.^18^ For IOP, its high relative attenuation (RA = 0.65) agrees well with reports showing very short half-lives under suboxic and anoxic conditions.^16,18,19^

Among all the compounds analyzed, AMS and VEN showed the highest attenuation (RA of 0.85 and 0.98, respectively). Both compounds were predominantly cationic under the experimental conditions, with AMS being more hydrophilic than VEN (Figure 5). As previously discussed for MET and SIT, cations typically experience stronger sorption than neutral or anionic compounds due to electrostatic interactions with sediments.^12,41^ In the case of AMS, complete elution was observed in one of the columns fed with river water, while an initial breakthrough occurred in the two additional replicates, which translates into RM = 0.05. Moreover, low but quantifiable concentrations were detected in one of the CT replicates after 120 days of operation (Figure S21). These findings, together with the fact that AMS has been reported to show no significant removal in WWTPs,^59^ led us to conclude that strong sorption was the main attenuation pathway for AMS. For VEN, we observed a very small breakthrough in one of the replicates fed with river water (Figure S25), with one of them reaching a plateau, which is a typical breakthrough pattern for compounds undergoing both sorption and transformation.^60^ This behavior, together with previous studies showing that VEN can be biotransformed under anaerobic conditions,^61^ indicates that VEN was retarded by sorption but simultaneously biotransformed, with the latter appearing to be the predominant mechanism.

#### 3.3.5 Summary of Attenuation Behaviors

Overall, the analyzed TrOCs displayed a wide range of attenuation behaviors, (i) including transformation products formed from parent compounds (e.g., VSA), (ii) others showing almost conservative behavior (e.g., FCZ, CAN, and HCTZ), (iii) cases dominated by reversible sorption (e.g., CET, MET, SIT, TTA, CBZ, IRB, BTA, and AMS), and (iv) several exhibiting a different degree of anoxic biotransformation (e.g., GBP, DCF, DTA, IOP, SMX, OXP, and VEN). This variety of observed processes underlines how strongly speciation, polarity, and redox conditions control the fate of TrOCs.

To synthesize the observed behaviors across all investigated compounds, Table 3 provides a process-based classification distinguishing the relative contributions of sorption and biotransformation, as supported by the experimental observations discussed above.

**Table 3:** Interpretation of Compound Behavior during the Experiment^a^

TrOC	sorption	biotransformation	interpretation
VSA	0	+++	negligible sorption; known transformation product of parent compounds
TTA	++	0	moderate, largely reversible sorption; negligible biotransformation
CBZ	++	0	moderate, largely reversible sorption; persistent to biotransformation
FCZ	0	0	negligible sorption; no evidence of biotransformation (conservative behavior)
CAN	0	0	negligible sorption; no evidence of biotransformation (conservative behavior)
HCTZ	0	+	negligible sorption; low biotransformation
GBP	0	+	negligible sorption; low biotransformation
IRB	+	+	weak sorption; minor biotransformation
DCF	+	+	weak sorption; minor biotransformation
MET	+++	+	strong, largely reversible sorption; limited biotransformation
BTA	++	++	moderate sorption; moderate biotransformation
DTA	0	++	negligible sorption; moderate biotransformation
SIT	+++	++	strong, largely reversible sorption; moderate biotransformation
CET	+++	++	strong, largely reversible sorption; moderate biotransformation
SMX	0	+++	negligible sorption; strong biotransformation
IOP	0	+++	negligible sorption; strong biotransformation
OXP	0	+++	negligible sorption; strong biotransformation
AMS	+++	+	strong sorption; limited biotransformation
VEN	+	+++	weak sorption; strong biotransformation

a Symbols indicate the relative importance of each process: 0 = none/negligible, + = low, ++ = moderate, and +++ = high.

Although the classification in Table 3 provides a process-based overview, the attenuation behavior of several compounds, such as IOP, CET, HCTZ, GBP, AMS, DTA, OXP, and SMX, varied over time (Section S6). We link this to the temporal evolution of redox-sensitive species observed during the experiment (Figure 2), since biotransformation rates are commonly redox-dependent.^6,62^ At the same time, the applied flow rate resulted in a residence time of approximately 12 h, imposing a kinetic constraint on biotransformation. Under these conditions, compounds with slow transformation kinetics may appear persistent, even if they are ultimately biodegradable. Therefore, the reported RA and RM values represent cumulative descriptors that integrate both temporal variability and kinetic limitations, rather than constant process rates.

Furthermore, pronounced pH shifts were observed at the beginning of the experiment, especially in the river water columns, from an initial value of 7.5 to 7.9 after approximately 20 days (Figure S11). These changes reflect differences between field and experimental pH conditions (Figure S3). During this initial phase, concurrent changes were observed in the behavior of redox-sensitive species such as for SO_4_^2–^ (Figure 2c). Altogether, these observations indicate that microbial communities may have experienced temporary stress at the beginning of the experiment.

The comparison between river- and tap water-fed columns further indicates that biotransformation cannot be excluded in the tap water experiments. Despite the absence of TrOCs in the inflow, active redox conditions persisted, suggesting an ongoing microbial activity. This is particularly evident for compounds such as IOP, OXP, SMX, and DTA, which exhibited substantial attenuation in the river water-fed columns but were not eluted from the tap water columns, indicating removal within the sediment. In contrast, compounds such as CBZ, TTA, CET, MET, and SIT showed clear mobilization patterns, consistent with sorption-dominated but largely reversible behavior. These compounds are either relatively hydrophobic (high log D) or positively charged, supporting the importance of hydrophobic interactions and cation exchange. Finally, the variability observed among triplicates, for example, for CET, HCTZ, GBP, and AMS (Figure 5), suggests that small-scale heterogeneities in sediment composition and structure influenced attenuation processes.

### 3.4 Limitations of the Experimental Approach

While the column experiments provide mechanistic insights into TrOCs’ attenuation, several limitations should be considered when extrapolating the results to natural hyporheic systems. The experimental setup simplifies hydraulic conditions by imposing a one-dimensional, steady-flow regime, whereas natural hyporheic zones are characterized by three-dimensional flow paths, spatial heterogeneity, and transient hydrological dynamics. Although intact sediment cores were used to preserve the structure, some disturbance during sampling, transport, and installation cannot be fully excluded and may have affected the pore structure and microbial activity. A characterization of microbial communities at the beginning and at the end of the experiment would have helped to better constrain these potential effects. In addition, the use of acrylic columns may have introduced wall effects, potentially influencing the flow distribution and solute transport near the column boundaries. Furthermore, determination of sorbed masses at the beginning and at the end of the experiment could have improved the precision of the mass balance calculations, although this would have required the development of a sediment extraction method applicable to the broad range of compounds analyzed.

Further deviations from in situ conditions arise from the imposed experimental environment. In particular, sediments and associated microbial communities originating from a system with a pronounced seasonal temperature regime were exposed to nearly constant laboratory temperatures (20 °C) over several months. This may have altered microbial activity and transformation rates compared to field conditions, where temperature fluctuations influence both redox dynamics and biotransformation kinetics. While precautions were taken to minimize disturbance, including rapid installation under saturated conditions, some exposure of anoxic sediment layers to atmospheric oxygen cannot be entirely excluded. Finally, the results reflect conditions specific to the organic-rich sediments of the Landgraben stream, and attenuation patterns may differ in systems with different hydraulic, geochemical, and seasonal characteristics. For instance, additional column experiments using uncontaminated sediments flushed with contaminated river water or experiments under oxic conditions could provide further insights into attenuation processes under different environmental conditions. In this regard, future reactive transport modeling of the presented experiments could also contribute to a more mechanistic understanding of TrOC fate in the HZ.

### 3.5 Environmental Implications

The transport of TrOCs can be attenuated by biotransformation or sorption, with sorption largely resulting in retardation without net removal, as the compounds remain in the system, at least in natural environments.^15,18,38^ Also, it is widely accepted that the attenuation of TrOCs is promoted in oxic zones where biotransformation dominates.^6,21,48^ From this perspective, our study represents a more pessimistic case in which anoxic conditions prevail. We observed that more than half of the detected compounds in the river exhibited a RA below 25% over an exchange of hundreds of pore volumes. This suggests that polluted hyporheic environments with low hydraulic conductivity and a high content of particulate and dissolved organic matter have a limited capacity to attenuate many TrOCs, which may pose a risk to connected groundwater systems.

Furthermore, the reversible character of sorption, observed in our experiment for several compounds, underscores that even if stream concentrations decrease due to improved WWTP performance, there remains a high potential for the remobilization of TrOCs from hyporheic sediments. This highlights the need not only to improve the quality of surface waters but also to consider remediating contaminated riverbeds.

This study confirms that laboratory studies are a valuable approach for understanding the fate of TrOCs and that additional experiments under controlled conditions could help explain the behavior of other compounds and processes. However, we acknowledge that important characteristics of natural hyporheic environments must be investigated directly in the field. Further work should also focus on monitoring the attenuation of TrOCs not only in the riverbed sediments but also in the surrounding groundwater. Ultimately, a reliable assessment of TrOC transport in hyporheic and connected groundwater systems depends on understanding how redox conditions, sediment properties, and exchange processes jointly control their mobility and transformation.

## Supplementary Material



## Data Availability

Data are available from the corresponding author upon request.

## Notes

Environmental conditions at the sampling site; sediments composition; physicochemical properties of selected TrOCs; TrOC quantification; evolution of DOC, main electron acceptors and pH; and BCs of TrOCs (PDF)
