# Supplementary material for: Fate of Trace Organic Contaminants in the Hyporheic Zone: A Laboratory-Scale Column Experiment
Source: Environ Sci Technol. 2026 Jun 16;60(25):18093–104. doi: 10.1021/acs.est.5c18582 (PMC13325848; doi:10.1021/acs.est.5c18582)
Supplement: Supplementary file 1 [file es5c18582_si_001.pdf]

Supporting information for: Fate of Trace Organic Contaminants in the Hyporheic Zone: A Laboratory-Scale Column Experiment

Edinsson Muñoz-Vega,[*,†] Selina Hillmann,[†,‡] Mohammad Sajjad Abdighahroudi,[¶] Kai Ihle,[†] Carolin Bertold,[§] Christoph Schüth,[†,‖] Holger V. Lutze,[¶,⊥,#] and Stephan Schulz[†]

†*Institute of Applied Geosciences, Technical University of Darmstadt, 64287 Darmstadt, Germany*

‡*Hessian Agency for Nature Conservation, Environment and Geology (HLNUG), Groundwater, 65203 Wiesbaden, Germany*

¶*Chair of Environmental Analytics and Pollutants, Institute IWAR, Technical University of Darmstadt, 64287 Darmstadt, Germany*

§*Department Evolutionary Ecology & Environmental Toxicology, Goethe University Frankfurt, 60438 Frankfurt am Main, Germany*

‖*Water Resources Management Division, IWW Water Centre, 45476 Mülheim an der Ruhr, Germany*

⊥*Centre for Water and Environmental Research (ZWU), 45141 Essen, Germany*

#*Kompetenzzentrum Wasser Hessen, 60438 Frankfurt Am Main, Germany*

E-mail: edinsson.munoz@tu-darmstadt.de

This document contains 29 pages and the following content:

Sections S1 to S6

Figures S1 to S26

Tables S1 to S12

Contents

S1 Environmental and hydrochemical conditions at the sampling site S3

S2 Sediments composition S8

S3 Physicochemical properties of selected TrOCs S10

S4 TrOCs quantification S11

S5 Evolution of Dissolved Organic Carbon, main electron acceptors and pH S18

S6 Breakthrough curves TrOCs S22

S1 Environmental and hydrochemical conditions at the sampling site

The Landgraben is a small, lowland stream located in the Hessian Ried (Hesse, Germany), a flat alluvial plain characterized by intensive agricultural use and strong groundwater–surface water interactions. Riverbed cores and surface water samples used as inflow for the column experiments were collected near the city of Trebur (Figure S1).

Two monitoring stations operated by the Hesse State Office for Nature Conservation, Environment, and Geology (HLNUG) are located in the vicinity of the sampling site: a surface water station (ID 109), approximately 1.2 km upstream, and a groundwater monitoring well (ID 17691) located approximately 300 m from the sampling site (Figure S1).

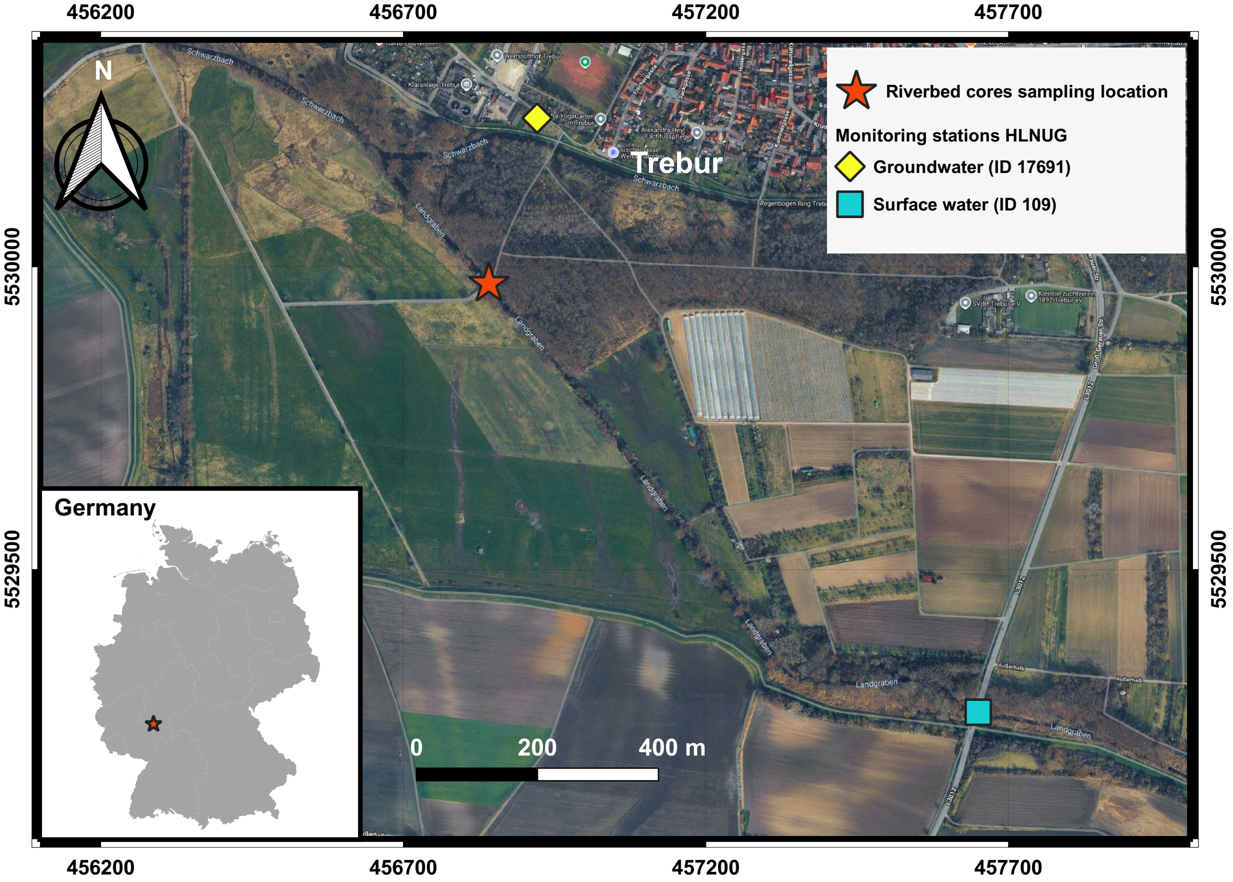

Figure S1: Location of monitoring stations (HLNUG dataset). Coordinate Reference System: WGS 84 / UTM zone 32N.

Surface water samples are collected monthly, whereas groundwater samples are obtained during less frequent monitoring campaigns. Data on physicochemical parameters, including TrOCs and major ions, are publicly available via the HLNUG "Wasserviewer" web portal.[1] Tables S1 and S2 summarize TrOCs for the surface water station (2024) and for the groundwater monitoring well (2023 to 2025), respectively, while Tables S3 and S4 present corresponding data of major ions for both stations.

Table S1: Summary statistics of TrOCs measured in this study at surface water station ID 109 in the year 2024. All values in μg/L. n = number of samples; LOD = limit of detection. Values below the LOD were substituted with the respective LOD.

Compound	Min	Max	Median	n	LOD
Diatrizoic Acid	0.33	2.90	1.50	12	0.05
Amisulpride	0.03	0.34	0.18	12	0.02
Atenolol	0.02	0.02	0.02	12	0.02
1,2,3-Benzotriazole	0.20	1.30	0.48	12	0.02
Candesartan	0.91	2.70	1.45	12	0.02
Carbamazepine	0.18	0.47	0.35	12	0.02
Cetirizine	0.51	1.60	0.78	12	0.02
Diclofenac	0.55	1.70	1.00	12	0.02
Fluconazole	0.02	0.14	0.04	12	0.02
Gabapentin	0.24	0.51	0.36	12	0.03
Hydrochlorothiazide	0.42	1.60	0.57	12	0.02
Iopromide	0.68	4.70	2.15	12	0.05
Irbesartan	0.07	0.15	0.12	12	0.02
Metoprolol	0.11	0.34	0.21	12	0.02
Oxipurinol	2.20	9.10	5.90	12	0.05
Sitagliptin	0.34	1.20	0.63	12	0.02
Sulfamethoxazole	0.09	0.26	0.12	12	0.02
Valsartan Acid	0.59	2.10	0.82	12	0.02
Venlafaxine	0.06	0.19	0.11	12	0.02

In terms of temperature, a Solinst Levelogger 5 equipped with a temperature sensor was installed in the river at the same location where the riverbed cores were collected. Figure S2 shows the temporal evolution of river temperature from 15.09.2024 to 15.09.2025. As a reference, air temperature recorded at the German Weather Service (DWD) station ID 917 in Darmstadt is also included in Figure S2.

[1] `https://umweltdaten.hessen.de/mapapps/resources/apps/wasserviewer/index.html?lang=en`

Table S2: Summary statistics of TrOCs measured in this study at groundwater monitoring station ID 17691 over the years 2023 to 2025. The well is screened between 7 and 11 m b.g.l. All values in μg/L. n = number of samples; LOD = limit of detection. Values below the LOD were substituted with the respective LOD.

Compound	Min	Max	Median	n	LOD
Diatrizoic Acid	0.10	0.23	0.10	3	0.10
Carbamazepine	0.25	0.39	0.27	3	0.05
Diclofenac	0.23	0.45	0.29	3	0.05
Iopromide	0.10	0.10	0.10	3	0.10
Sulfamethoxazole	0.05	0.05	0.05	3	0.05

Table S3: Summary statistics of selected physicochemical parameters at surface water station ID 109 in the year 2024. All concentrations are reported in mM. DOC was converted assuming 12 g/mol. n = number of samples. n.a. = not available.

Compound	Min	Max	Mean	n
O_2	0.1630	0.3560	0.2560	12
DOC	n.a.	n.a.	n.a.	n.a.
NO_3^-	0.0640	0.1640	0.1060	13
Fe	0.0004	0.0009	0.0006	13
Mn	0.0004	0.0026	0.0011	14
SO_4^{2-}	0.5530	1.5610	1.0150	12

Table S4: Summary statistics of selected physicochemical parameters at groundwater monitoring station ID 17691 over the years 2023 to 2025. The well is screened between 7 and 11 m b.g.l. All concentrations are reported in mM. DOC was converted assuming 12 g/mol. n = number of samples.

Compound	Min	Max	Mean	n
O_2	0.0009	0.0125	0.0053	5
DOC	0.3170	0.3750	0.3380	4
NO_3^-	0.0002	0.0900	0.0370	4
Fe	0.0005	0.0115	0.0007	4
Mn	0.0038	0.0062	0.0051	4
SO_4^{2-}	0.9890	1.5610	1.1790	4

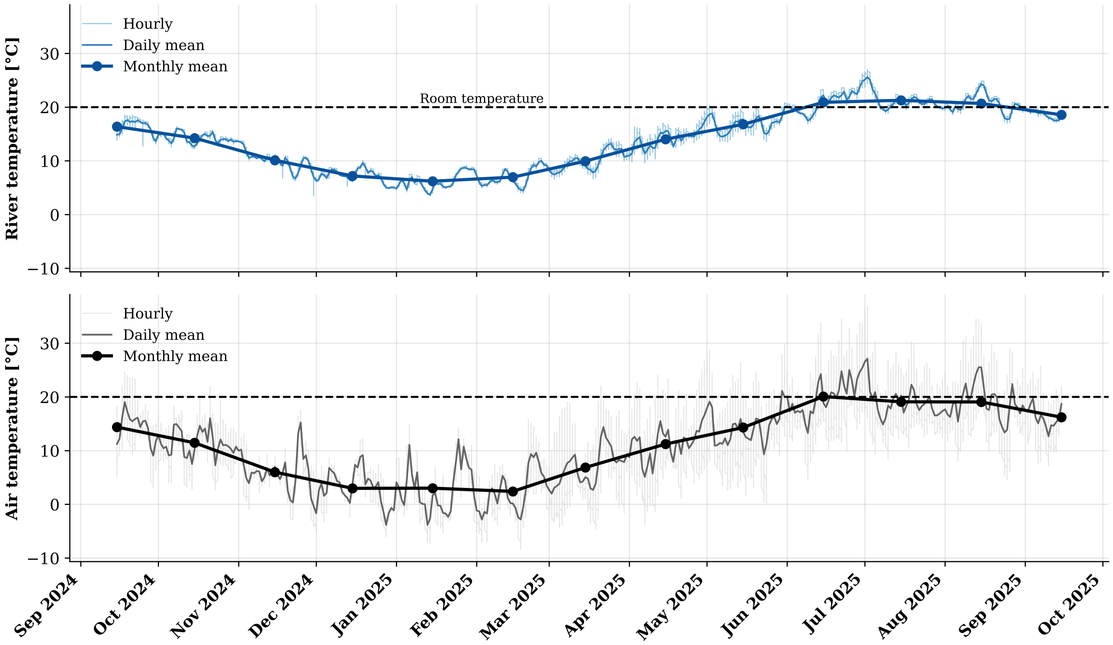

Figure S2: Temporal evolution of river water temperature at the place where the riverbed cores were collected and air temperature in Darmstadt.

Furthermore, Figure S3 presents pH measurements of river water obtained directly in the field and under laboratory conditions.

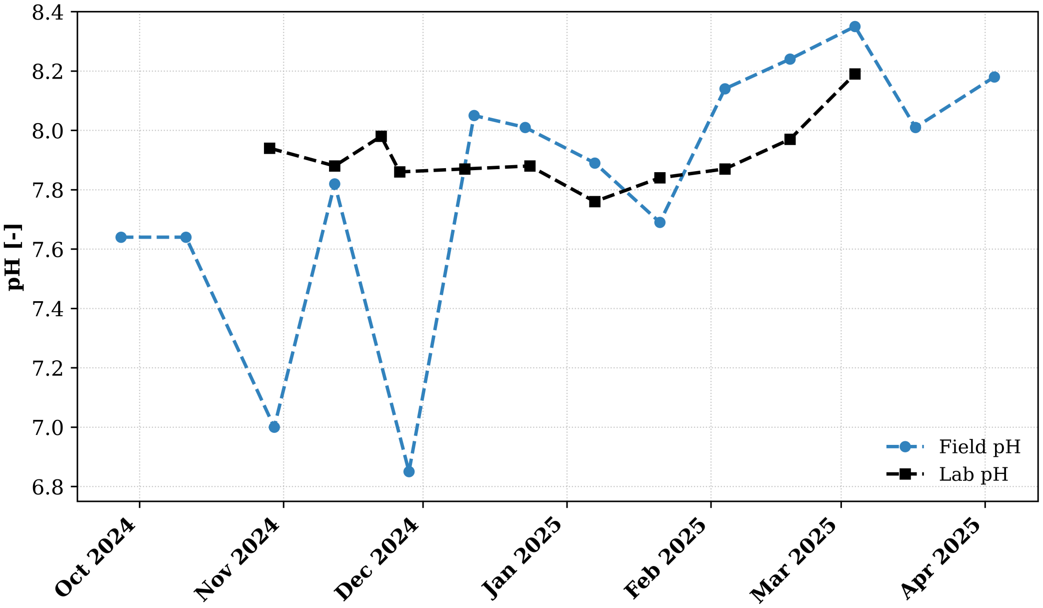

Figure S3: pH of river water measured in the field and under laboratory conditions. Field measurements do not necessarily correspond to the water used in the column experiments.

To assess the evolution of redox potential under field conditions in the riverbed, an in situ oxidation–reduction potential probe (Paleo Terra, Netherlands) was vertically installed together with an AgCl (3 M KCl) reference electrode to measure Eh at three depths (5, 15, and 25 cm). Measurements were initiated on 15 March 2026 and recorded at 30 minute intervals. The average ambient temperature during the week shown in Figure S4 was 8.1°C, which is comparable to the daily mean temperature of 10.8°C on 28 October 2024, when the sediment cores were collected.

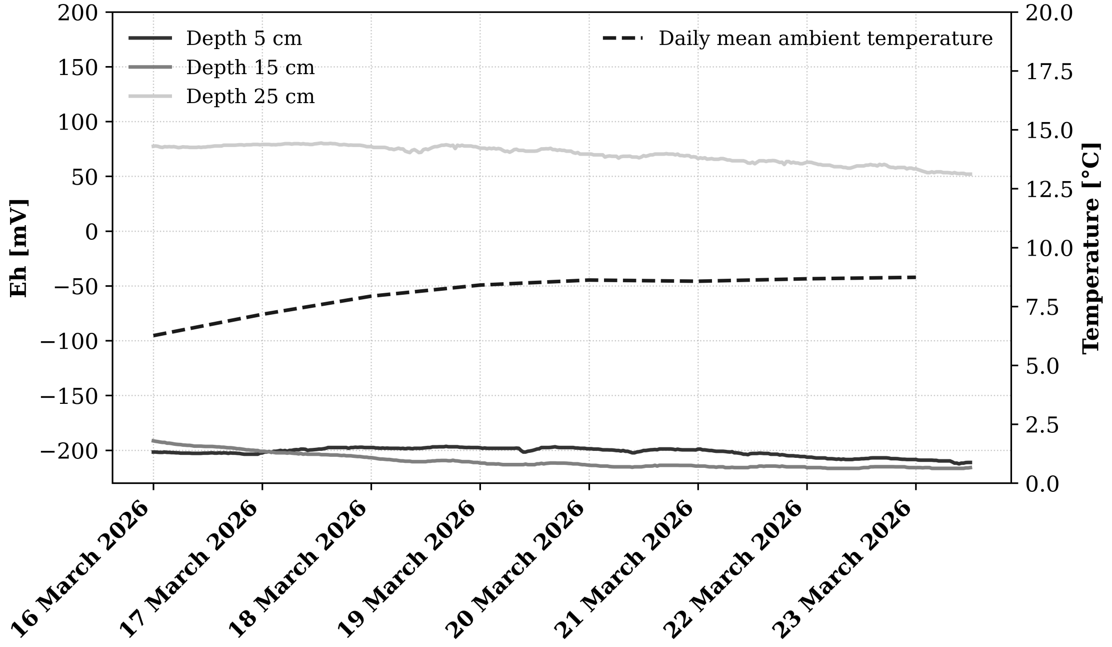

Figure S4: Eh at the riverbed of the Landgraben stream.

S2 Sediments composition

Table S5: Major oxide composition of the riverbed sediments at different depths after Loss on Ignition, determined by X-Ray fluorescence (XRF). Values are reported as averages with standard deviations from five samples.

Oxide	Depth 0 -10 cm [%]	Depth 10 -20 cm [%]	Depth 20 - 30 cm [%]
SiO_2	83.9 ± 1.4	77.9 ± 1.9	82.7 ± 2.7
TiO_2	0.2 ± 0.0	0.3 ± 0.0	0.2 ± 0.1
Al_2O_3	5.6 ± 0.4	7.8 ± 0.7	6.3 ± 0.8
Fe_2O_3	1.3 ± 0.2	2.7 ± 0.4	1.9 ± 0.6
MnO	0.0 ± 0.0	0.1 ± 0.0	0.0 ± 0.0
MgO	0.5 ± 0.1	1.1 ± 0.2	0.7 ± 0.2
CaO	5.2 ± 1.2	6.2 ± 0.4	4.6 ± 0.8
Na_2O	1.2 ± 0.0	1.4 ± 0.0	1.2 ± 0.1
K_2O	2.0 ± 0.0	2.1 ± 0.1	2.0 ± 0.0
P_2O_5	0.2 ± 0.0	0.4 ± 0.2	0.3 ± 0.2

Table S6: Physicochemical properties of riverbed sediments for individual samples obtained from additional cores (ACo) collected at the study site.

Parameter	Depth 0 - 10 cm	Depth 10 - 20 cm	Depth 20 - 30 cm
Mean particle size [mm] (ACo1)	0.15	0.18	0.19
Mean particle size [mm] (ACo2)	0.15	0.15	0.10
Mean particle size [mm] (ACo3)	0.14	0.14	0.13
Mean particle size [mm] (ACo4)	0.15	0.15	0.16
Mean particle size [mm] (ACo5)	0.15	0.21	0.08
Organic matter content [%] (ACo1)	3.94	7.82	7.64
Organic matter content [%] (ACo2)	3.70	5.65	2.66
Organic matter content [%] (ACo3)	2.92	7.27	8.57
Organic matter content [%] (ACo4)	5.22	11.80	5.83
Organic matter content [%] (ACo5)	3.78	6.55	4.89
Organic carbon content [%] (ACo1)	1.07	2.46	2.48
Organic carbon content [%] (ACo2)	1.01	1.49	0.71
Organic carbon content [%] (ACo3)	0.74	2.05	2.66
Organic carbon content [%] (ACo4)	1.50	4.44	1.61
Organic carbon content [%] (ACo5)	1.01	1.85	1.37
CEC [cmol+/kg] (ACo1)	4.94	10.15	7.75
CEC [cmol+/kg] (ACo2)	6.55	10.15	3.49
CEC [cmol+/kg] (ACo3)	4.49	16.21	9.47
CEC [cmol+/kg] (ACo4)	6.11	7.54	8.04
CEC [cmol+/kg] (ACo5)	7.47	6.70	14.82

S3 Physicochemical properties of selected TrOCs

Table S7: Physicochemical properties of TrOCs analyzed in the column experiments. Properties at pH = 7.8 were estimated with Chemaxon calculators.

Compound	Code	Substance Class	MW [g/mol]	Acidic pKa / Basic pKa	log P	log D (pH = 7.8)	Species and Percentage (pH = 7.8)
**Neutral Compounds**							
1,2,3-Benzotriazole	BTA	Corrosion inhibitor	119.1	8.63 / 0.58	1.34	1.24	-1 / 0 | (13/87)
Carbamazepine	CBZ	Anticonvulsant	236.2	15.96 / No	2.67	2.77	0
Cetirizine	CET	Histamine	388.8	2.97 / 7.97	2.17	0.68	-1 / 0 | (40/60)
Ciprofloxacin	CIP	Antibiotic	331.3	5.96 / 8.69	0.65	-0.78	-1 / 0 / +1 | (1/87/12)
Fluconazole	FCZ	Antifungal agent	306.2	12.68 / 2.87	0.5	0.56	0
Gabapentin	GBP	Anticonvulsant	171.2	4.63 / 9.91	1.19	-1.27	-1 / 0 | (1/99)
Hydrochlorothiazide	HCTZ	Antihypertensive agent	297.7	9.6 / -1.53	-0.07	-0.58	-1 / 0 | (2/98)
Iopromide	IOP	X-ray contrast agent	791.1	11.12 / 0.38	-2.95	-0.45	0
Tolyltriazole	TTA	Corrosion inhibitor	133.1	8.93 / 0.76	1.80	1.78	-1 / 0 | (7/93)
Ritalinic Acid	RA	Antipsychotic agent	219.2	3.75 / 10.08	2.08	-0.36	-1 / 0 | (1/99)
**Anionic Compounds**							
Candesartan	CAN	Antihypertensive agent	440.4	3.92 / 2.07	5.01	-0.19	-2
Diatrizoic Acid	DTA	X-ray contrast agent	613.9	2.17 / No	0.45	-0.63	-1
Diclofenac	DCF	Anti-Inflammatory agent	296.1	4.01 / -0.58	4.06	0.92	-1
Irbesartan	IRB	Antihypertensive agent	428.5	4.23 / 1.23	4.5	3.34	-1
Sulfamethoxazole	SMX	Antibiotic	253.2	5.86 / 1.97	0.89	-0.12	-1 / 0 | (99/1)
Oxipurinol	OXP	Antihypertensive agent metabolite	152.1	6.45 / -0.1	-1.35	-0.85	-2 / -1 / 0 | (14/82/4)
Valsartan Acid	VSA	Antihypertensive agent metabolite	266.2	4.03 / -1.56	3.18	-1.6	-2 / -1 | (99/1)
**Cationic Compounds**							
Amisulpride	AMS	Antipsychotic agent	369.4	13.61 / 8.88	1.60	-0.86	0 / +1 | (8/92)
Atenolol	ATN	Antihypertensive agent	266.3	14.08 / 9.27	0.1	-1.05	0 / +1 | (3/97)
Metoprolol	MET	Antihypertensive agent	267.3	14.09 / 9.27	1.79	0.28	0 / +1 | (3/97)
Sitagliptin	SIT	Antidiabetic	407.3	No / 8.66	1.30	0.34	0 / +1 | (12/88)
Venlafaxine	VEN	Antipsychotic agent	277.4	14.42 / 9.01	2.91	1.50	0 / +1 | (6/94)

S4 TrOCs quantification

For the determination of TrOC concentrations, a mixed stock solution containing the 22 target TrOCs was prepared in methanol (MeOH) and used for the preparation of external calibration standards. The compounds used, together with their chemical formulas, CAS numbers, purities, and manufacturers, are listed in Table S8.

Table S8: Target trace organic contaminants (TrOCs) used for preparation of the mixed stock solution for external calibration standards.

Compound	Chemical formula	CAS number	Purity	Manufacturer
1,2,3-Benzotriazole	$C_6H_5N_3$	95-14-07	98.0%	TCI Deutschland GmbH
Tolyltriazole	$C_7H_7N_3$	29385-43-1	98.0%	Abcr Germany
Amisulpride	$C_{17}H_{27}N_3O_4S$	71675-85-9	98.0%	TCI Deutschland GmbH
Atenolol	$C_{14}H_{22}N_2O_3$	29122-68-7	98.0%	TCI Deutschland GmbH
Candesartan	$C_{24}H_{20}N_6O_3$	139481-59-7	97.0%	Abcr Germany
Carbamazepine	$C_{15}H_{12}N_2O$	298-46-4	98.0%	Sigma-Aldrich
Cetirizine dihydrochloride	$C_{21}H_{25}ClN_2O_3 \cdot 2HCl$	83881-52-1	98.0%	Sigma-Aldrich
Ciprofloxacin	$C_{17}H_{18}FN_3O_3$	85721-33-1	98.0%	TCI Deutschland GmbH
Diatrizoic Acid	$C_{11}H_9I_3N_2O_4$	117-96-4	99.0%	Alfa Aesar
Diclofenac sodium	$C_{14}H_{10}Cl_2NNaO_2$	15307-79-6	98.0%	Sigma-Aldrich
Fluconazole	$C_{13}H_{12}F_2N_6O$	86386-73-4	98.0%	Abcr Germany
Gabapentin	$C_9H_{17}NO_2$	60142-96-3	97.5%	Thermo Scientific Chemicals
Hydrochlorothiazide	$C_7H_8ClN_3O_4S_2$	58-93-5	97.0%	TCI Deutschland GmbH
Iopromide	$C_{18}H_{24}I_3N_3O$	73334-07-3	98.0%	TCI Deutschland GmbH
Irbesartan	$C_{25}H_{28}N_6O$	138402-11-6	98.0%	TCI Deutschland GmbH
Metoprolol tartrate	$C_{15}H_{25}NO_3 \cdot 0.5C_4H_6O_6$	56392-17-7	98.0%	TCI Deutschland GmbH
Oxipurinol	$C_5H_4N_4O_2$	2465-59-0	98.0%	Abcr Germany
Ritalinic Acid	$C_{13}H_{17}NO_2$	19395-41-6	98.0%	TCI Deutschland GmbH
Sitagliptin	$C_{16}H_{15}F_6N_5O$	486460-32-6	95.0%	Abcr Germany
Sulfamethoxazole	$C_{10}H_{11}N_3O_3S$	723-46-6	98.0%	Sigma-Aldrich
Valsartan Acid	$C_{14}H_{10}N_4O_2$	164265-78-5	95.0%	TRC (LGC Standards)
Venlafaxine hydrochloride	$C_{17}H_{27}NO_2 \cdot HCl$	93413-69-5	98.0%	TCI Deutschland GmbH

Mass spectrometry was performed using an Agilent 6470 Triple Quad system in Dynamic Multiple Reaction Monitoring (DMRM) mode, hyphenated with an Agilent 1260 Infinity II for chromatographic separation. Agilent MassHunter was used for data acquisition and analysis. An Agilent Poroshell 120 (EC-C18 2.7μm, 3×150 mm) column with a gradient

elution (Table S9) by ultra pure water plus 0.1% formic acid and acetonitrile plus 0.1% formic acid was utilized for chromatography. Flow rate was set at 0.5 mL/min and injection volume was 20 μL. LC-MS grade water, acetonitrile, and formic acid were purchased from VWR chemicals. The source parameters are shown in Table S10. The DMRM parameters, including both transition ions, are shown in Table S11.

Table S9: Gradient elution of the chromatographic method.

**Time (min)**	**Water (%)**	**Acetonitrile (%)**
0.00	95	5
3.00	95	5
9.00	60	40
15.00	10	90
20.00	10	90
20.01	95	5
26.00	95	5

Table S10: Ion source parameters.

**Parameter**	**Value**
Gas Temperature [°C]	300
Gas Flow [L/min]	10
Nebulizer [psi]	45
Sheath Gas Temperature [°C]	225
Sheath Gas Flow [L/min]	11
Capillary Voltage [V]	3500

Table S11: DMRM parameters.

Compound	RT [min]	ESI mode	Fragmentor [V]	Precursor Ion [m/z]	Collision Energy [eV]	Product Ion [m/z]
1,2,3-Benzotriazole†	8.86	Pos	98	120	25	65
1,2,3-Benzotriazole†	8.86	Pos	98	120	45	39
Tolyltriazole†	10.38	Pos	94	134.1	29	77.1
Tolyltriazole†	10.38	Pos	94	134.1	53	51.1
Amisulpride	8.87	Pos	134	370.2	29	242
Amisulpride	8.87	Pos	134	370.2	49	195.9
Atenolol	6.48	Pos	109	267.2	29	145
Atenolol	6.48	Pos	109	267.2	33	56.2
Candesartan	13.16	Pos	101	441.2	9	263.1
Candesartan	13.16	Pos	101	441.2	29	192
Carbamazepine	12.55	Pos	118	237.1	21	194
Carbamazepine	12.55	Pos	118	237.1	41	179.1
Cetirizine	12.49	Pos	99	389.2	25	201
Cetirizine	12.49	Pos	99	389.2	50	166.1
Ciprofloxacin	8.87	Pos	119	332.1	17	288.1
Ciprofloxacin	8.87	Pos	119	332.1	29	245.1
Diatrizoic Acid	3.63	Pos	105	614.8	17	361
Diatrizoic Acid	3.63	Pos	105	614.8	45	233.1
Diclofenac	15.34	Pos	98	296	13	250

Continued on next page

Compound	RT [min]	ESI mode	Fragmentor [V]	Precursor Ion [m/z]	Collision Energy [eV]	Product Ion [m/z]
Diclofenac	15.34	Pos	98	296	21	215
Fluconazole	9.79	Pos	66	307.1	17	238
Fluconazole	9.79	Pos	66	307.1	17	220
Gabapentin	7.58	Pos	98	172.1	12	154.1
Gabapentin	7.58	Pos	98	172.1	16	137.1
Hydrochlorothiazide	8.19	Neg	147	295.9	21	269
Hydrochlorothiazide	8.19	Neg	147	295.9	25	205
Iopromide	7.10	Pos	160	791.9	25	572.9
Iopromide	7.10	Pos	160	791.9	61	299.9
Irbesartan	12.69	Neg	129	427.2	17	399.1
Irbesartan	12.69	Neg	129	427.2	29	193.1
Metoprolol	9.55	Pos	104	268.2	21	116
Metoprolol	9.55	Pos	104	268.2	25	74.1
Oxypurinol	2.28	Neg	87	151	21	107.6
Oxypurinol	2.28	Neg	87	151	22	42.1
Ritalinic Acid	8.92	Pos	103	220.1	25	84.1
Ritalinic Acid	8.92	Pos	103	220.1	50	56.2
Sitagliptin	10.10	Pos	109	408.1	21	235
Sitagliptin	10.10	Pos	109	408.1	33	174
Sulfamethoxazole	10.76	Pos	103	254.1	17	156

Continued on next page

Compound	RT [min]	ESI mode	Fragmentor [V]	Precursor Ion [m/z]	Collision Energy [eV]	Product Ion [m/z]
Sulfamethoxazole	10.76	Pos	103	254.1	33	92.1
Valsartan Acid	11.47	Pos	109	267.1	17	206
Valsartan Acid	11.47	Pos	109	267.1	49	151
Venlafaxine	10.51	Pos	66	278.2	37	121
Venlafaxine	10.51	Pos	66	278.2	25	58.2

†Reported as the sum of all isomers.

For quantification of TrOCs, a single-point standard addition approach was implemented in this research. Every sample was analyzed twice, unspiked and spiked. For the spiked analysis, 50 μL of a mixed stock solution (6 μg/L of each TrOC) was added to 1 mL of each sample. With this approach, matrix effects and recovery were calculated for each sample individually. Analytical accuracy was ensured by mathematically correcting all data using the measured recovery from the standard addition. By using a low injection volume (20 μL) and a high flow rate (0.5 mL/min) in this method, the total mass of co-eluting matrix constituents entering the ion source was reduced and resulted in a low matrix effect for the majority of the measured compounds. Furthermore, procedural blanks analyzed every 10 injections confirmed the absence of carryover, and the consistent response of the spikes throughout the sequence served as a continuous control for instrumental drift and replicate precision. The average recoveries for all samples of each compound are presented in Table S12.

Calibration was based on a 9-point external calibration curve (0.02; 0.05; 0.1; 0.2; 0.5; 1; 2; 5 and 10 μg/L). Limits of quantification (LOQs) were defined as the lowest calibration points with signal-to-noise ratios greater than 10:1. These values are listed in Table S12, together with the maximum concentration used in the linear calibration (MOQ) for each substance and the corresponding coefficient of determination (R^2). All samples with calculated concentrations below the LOQ were set to zero for mass balance calculations. The limit of detection (LOD) was not used in this study.

Table S12: Analytical performance parameters for target compounds.

**Compound**	**LOQ [μg/L]**	**MOQ [μg/L]**	R^2	**Recovery [%, mean ± SD]**
1,2,3-Benzotriazole	0.02	5	0.9994	93 ± 15
Tolyltriazole	0.02	2	0.9999	86 ± 28
Amisulpride	0.02	1	0.9925	153 ± 11
Atenolol	0.02	0.5	0.9988	96 ± 7
Candesartan	0.02	5	0.9999	56 ± 5
Carbamazepine	0.02	1	0.9998	90 ± 17
Cetirizine	0.02	1	0.9979	84 ± 21
Ciprofloxacin	0.20	5	0.9940	117 ± 31
Diatrizoic Acid	0.02	2	1.0000	123 ± 37
Diclofenac*	0.02	2	0.9992	22 ± 6
Fluconazole	0.02	0.5	1.0000	85 ± 13
Gabapentin	0.02	1	1.0000	94 ± 7
Hydrochlorothiazide	0.02	2	0.9993	92 ± 27
Iopromide	0.05	5	0.9985	103 ± 18
Irbesartan	0.02	1	0.9996	76 ± 15
Metoprolol	0.02	1	0.9977	113 ± 8
Oxipurinol**	0.02	5	0.9984	21 ± 20
Ritalinic Acid	0.02	0.5	0.9884	88 ± 10
Sitagliptin	0.02	2	0.9985	91 ± 12
Sulfamethoxazole	0.02	0.5	0.9997	72 ± 22
Valsartan Acid	0.02	5	1.0000	77 ± 20
Venlafaxine	0.02	1	0.9941	129 ± 8

*Not corrected by recovery due to instability of Diclofenac in the spiking mixture.
**Not corrected by recovery due to the relatively low level of spiking concentration compared to the actual measured concentration.

S5 Evolution of Dissolved Organic Carbon, main electron acceptors and pH

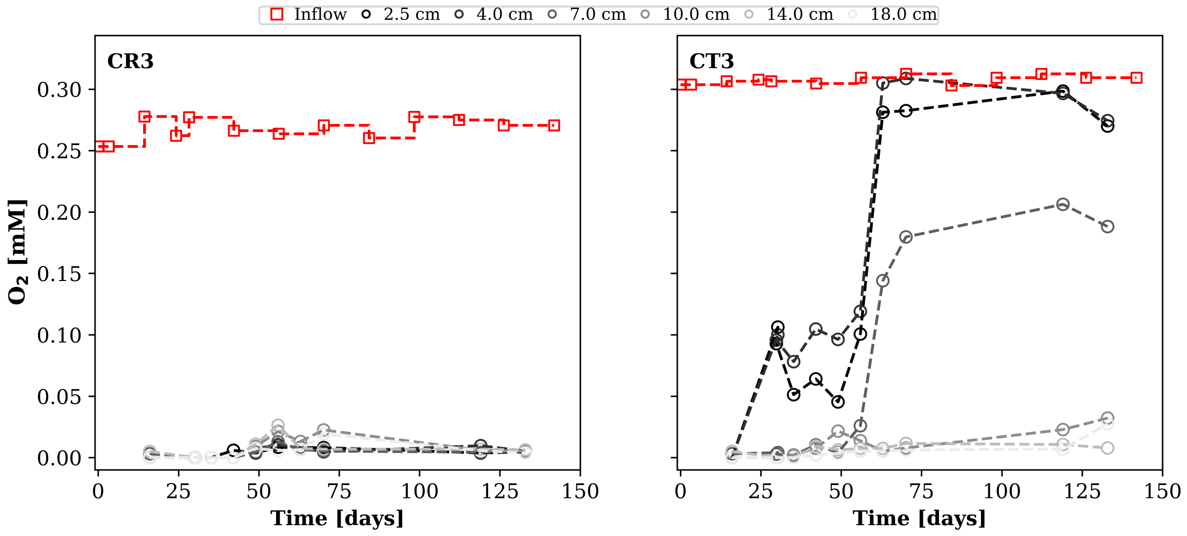

Figure S5: Evolution of Dissolved Oxygen in the columns equipped with oxygen spots. Values in cm refer to distances to the inflow section of each column.

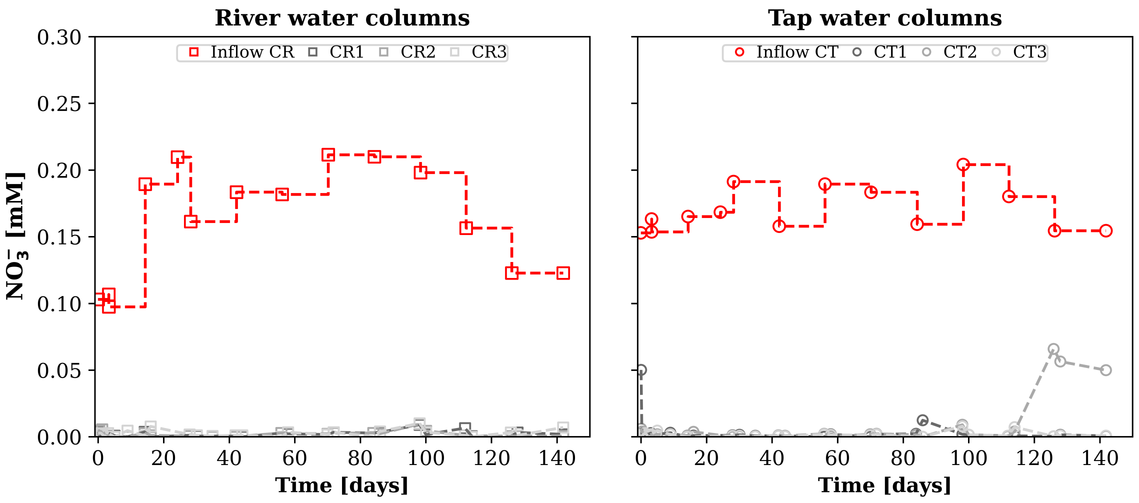

Figure S6: Evolution of Nitrate in the six columns.

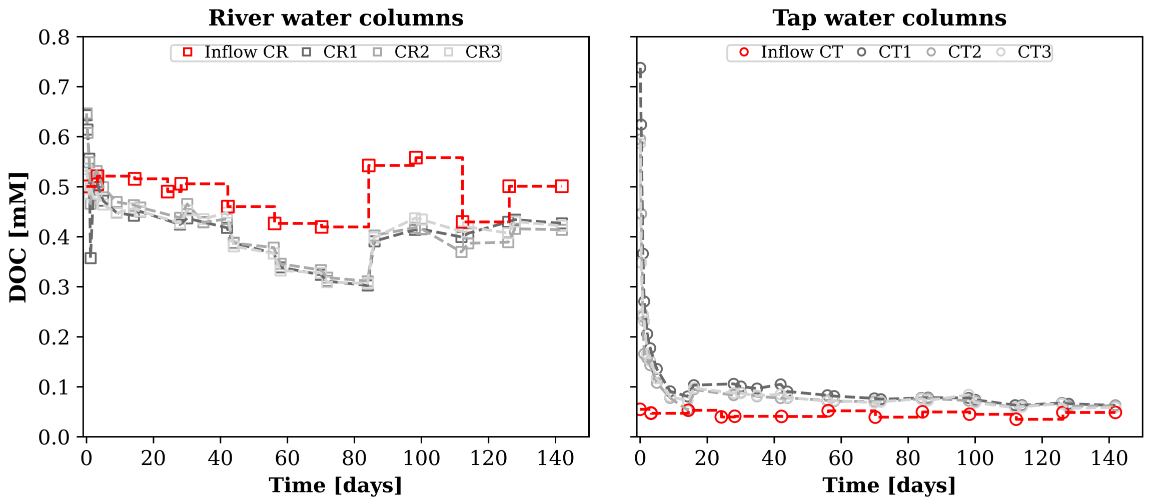

Figure S7: Evolution of Dissolved Organic Carbon in the six columns.

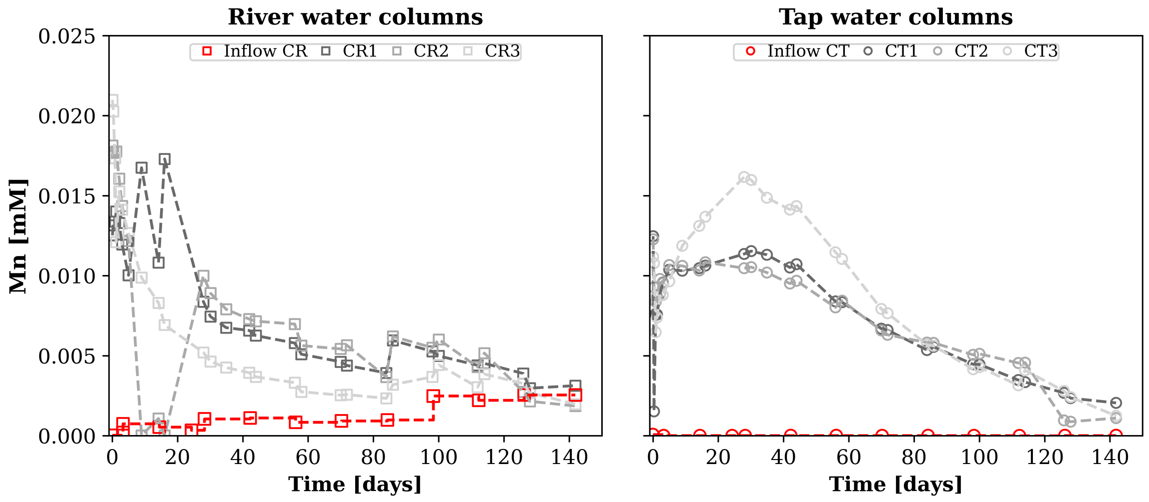

Figure S8: Evolution of Manganese in the six columns.

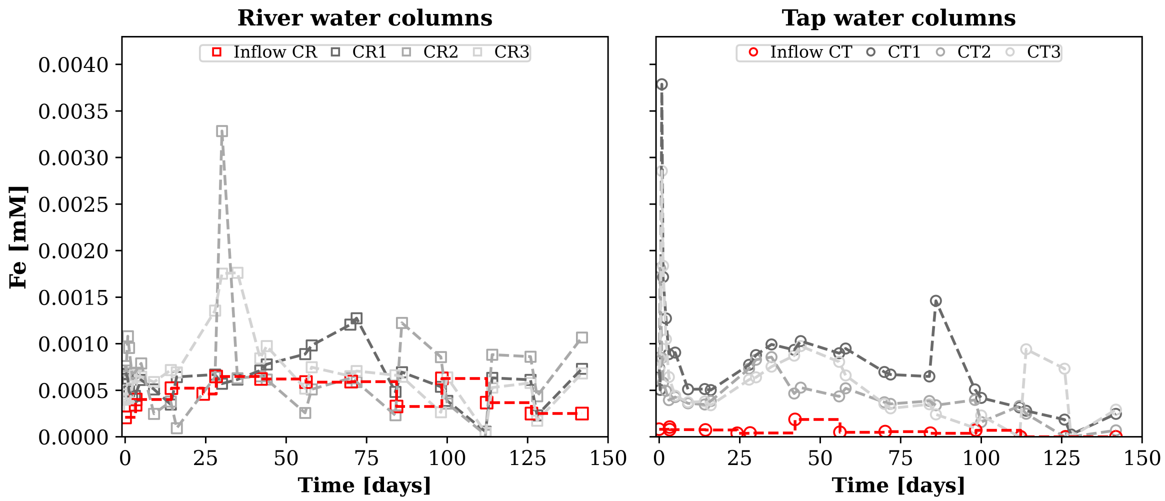

Figure S9: Evolution of Iron in the six columns.

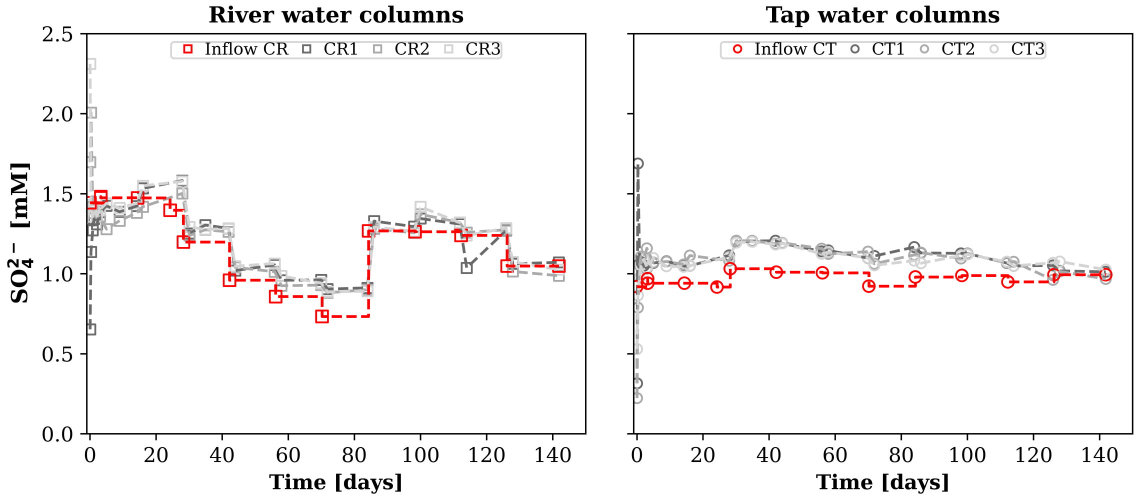

Figure S10: Evolution of Sulfate in the six columns.

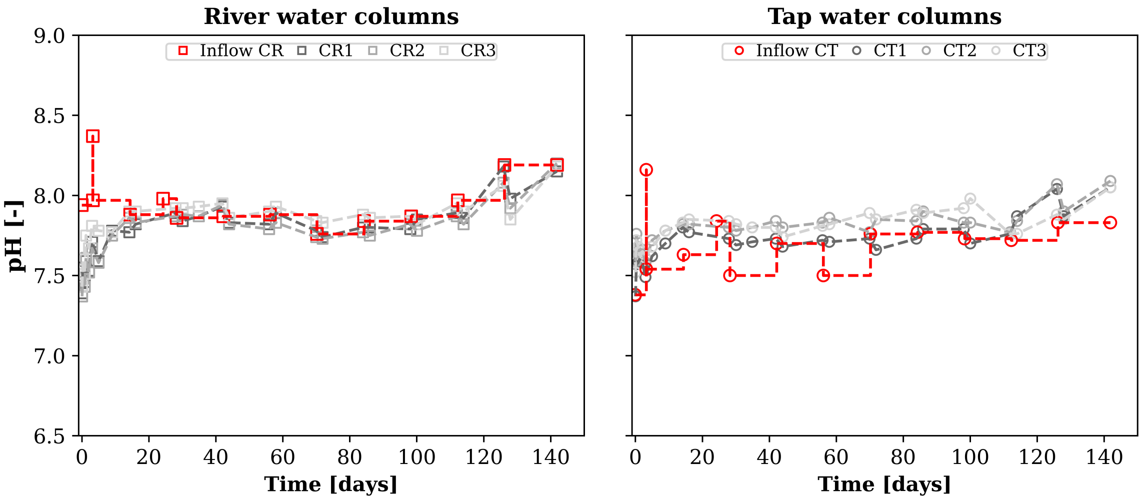

Figure S11: Evolution of pH in the six columns.

S6 Breakthrough curves TrOCs

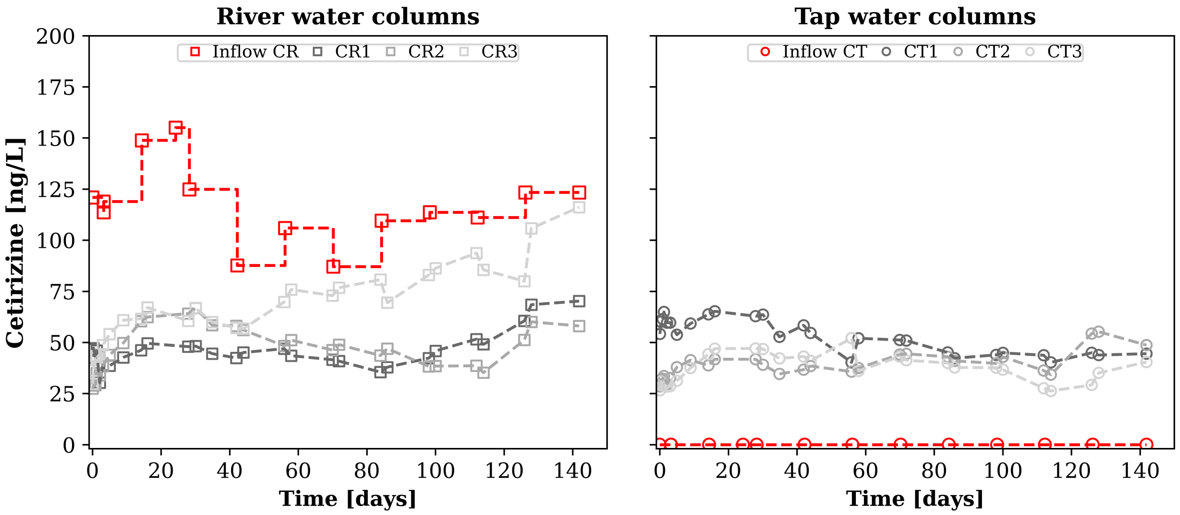

Figure S12: Evolution of Cetirizine in the six columns.

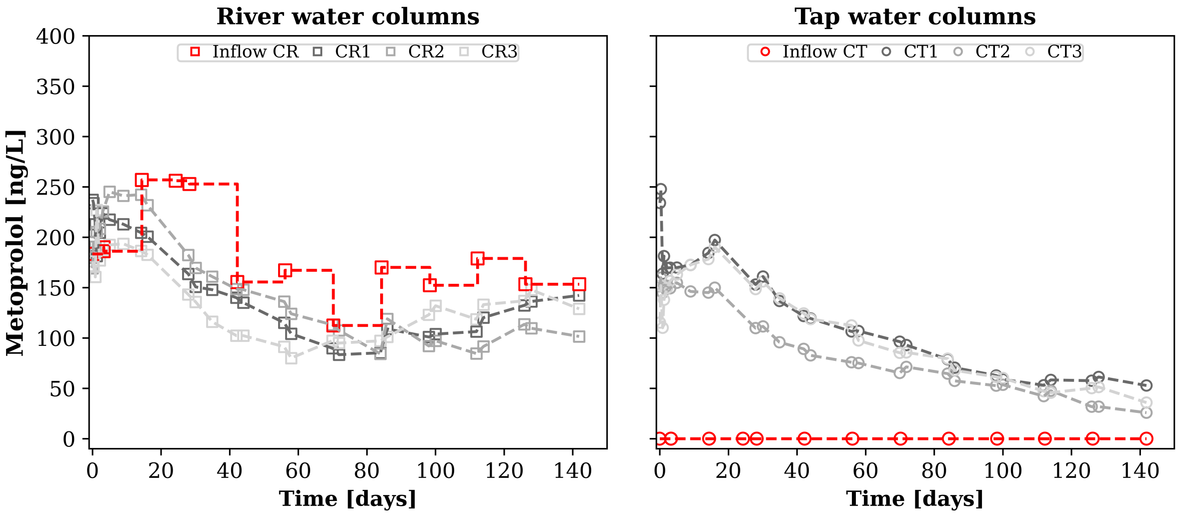

Figure S13: Evolution of Metoprolol in the six columns.

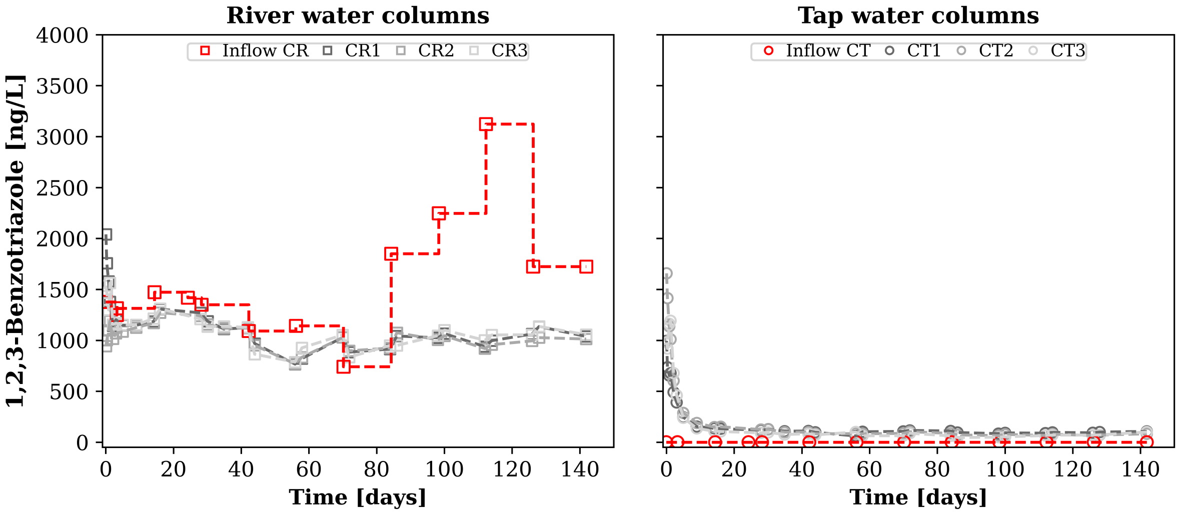

Figure S14: Evolution of 1,2,3-Benzotriazole in the six columns.

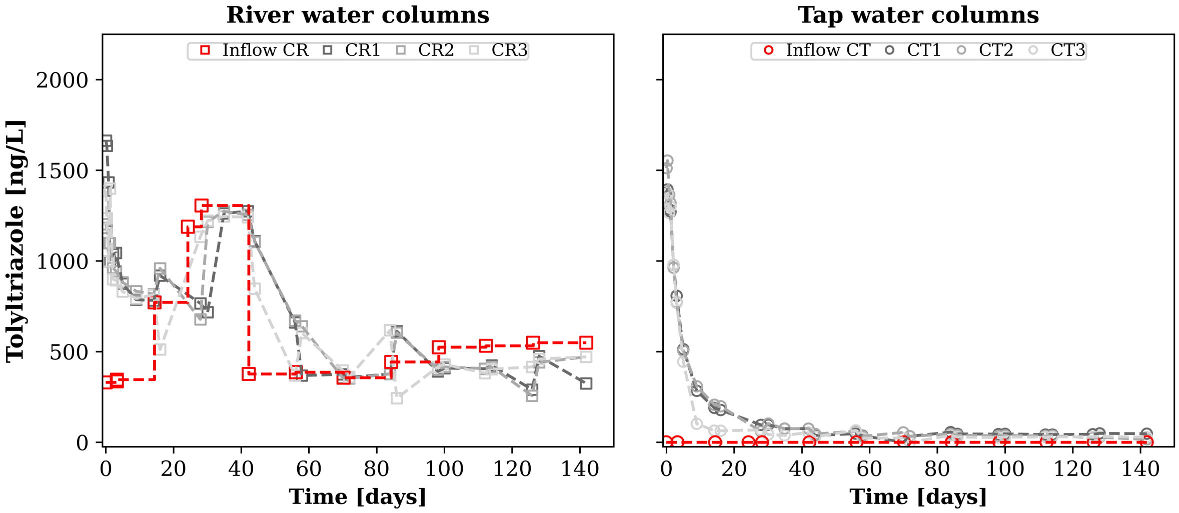

Figure S15: Evolution of Tolyltriazole in the six columns.

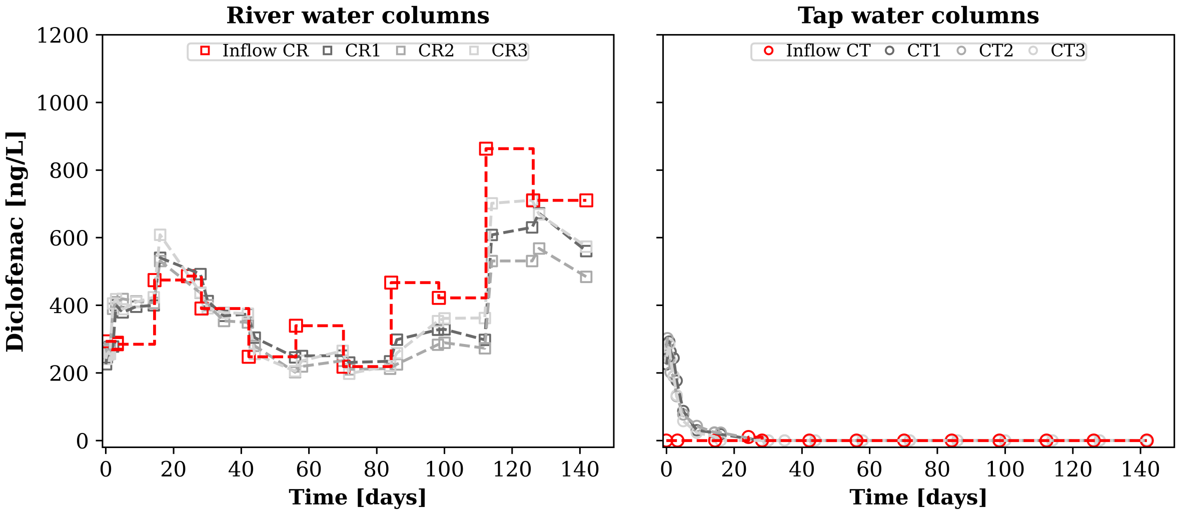

Figure S16: Evolution of Diclofenac in the six columns.

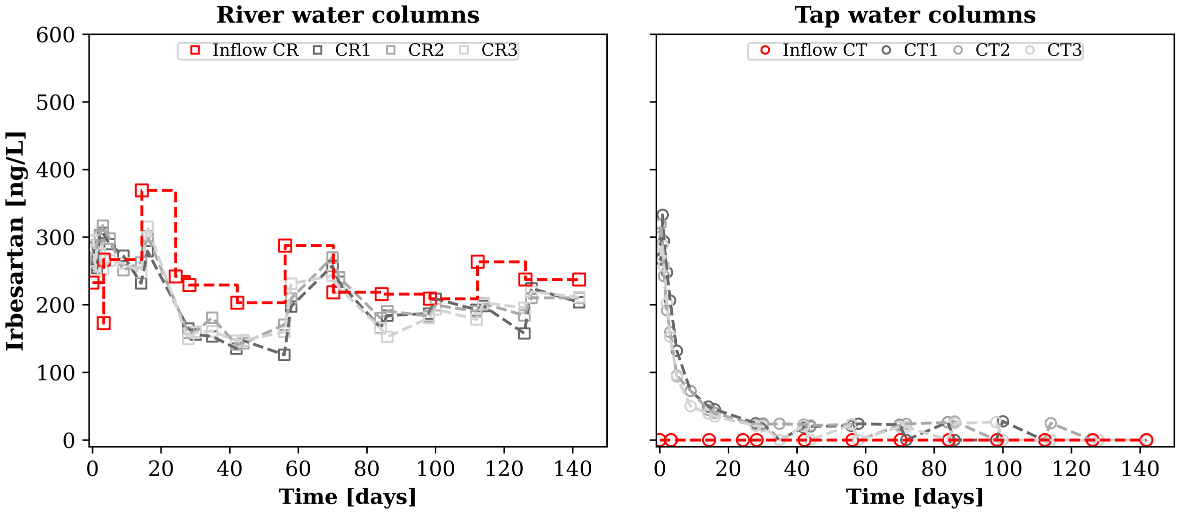

Figure S17: Evolution of Irbesartan in the six columns.

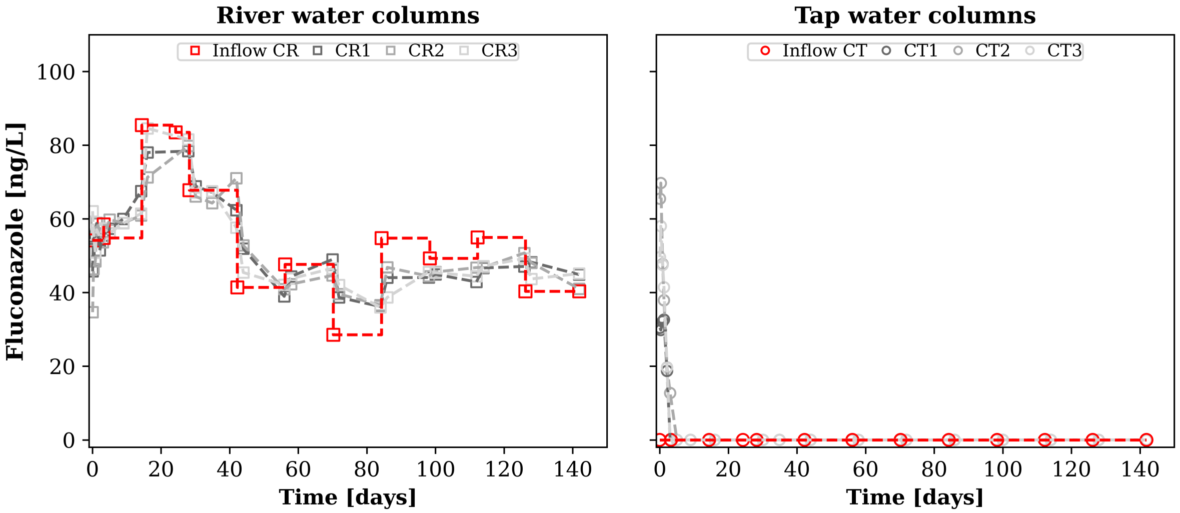

Figure S18: Evolution of Fluconazole in the six columns.

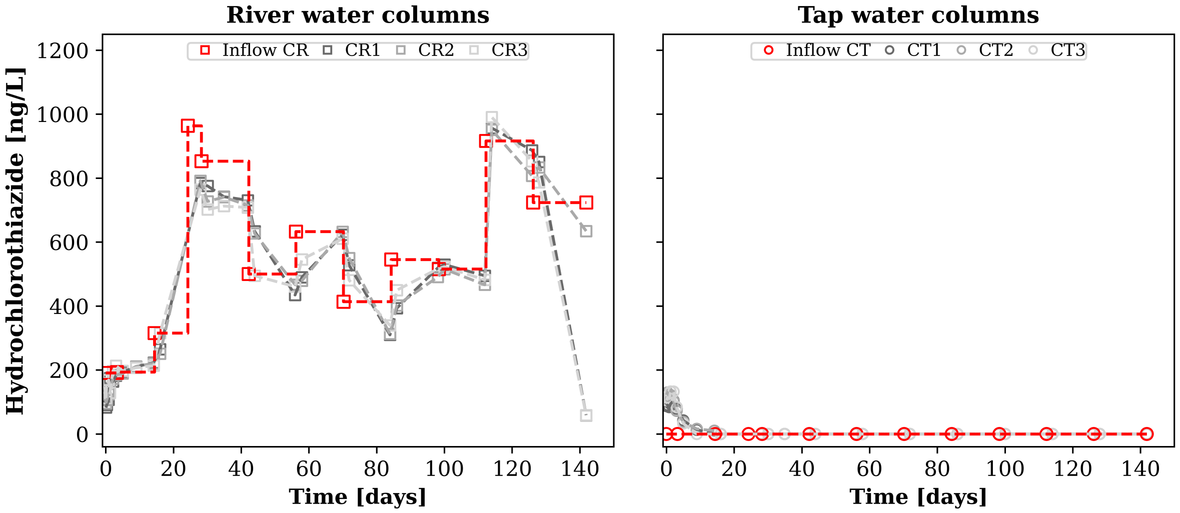

Figure S19: Evolution of Hydrochlorothiazide in the six columns.

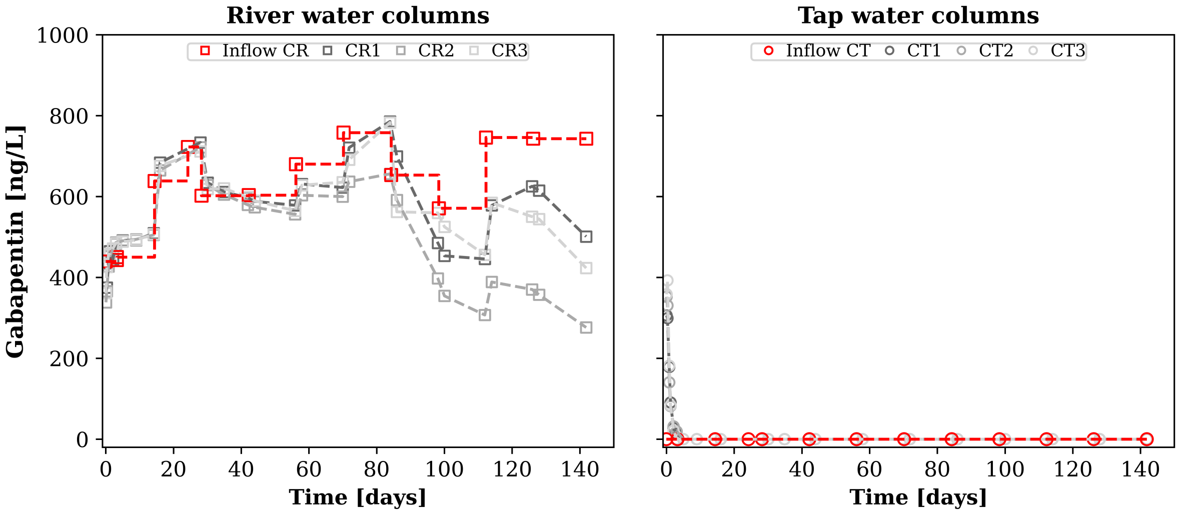

Figure S20: Evolution of Gabapentin in the six columns.

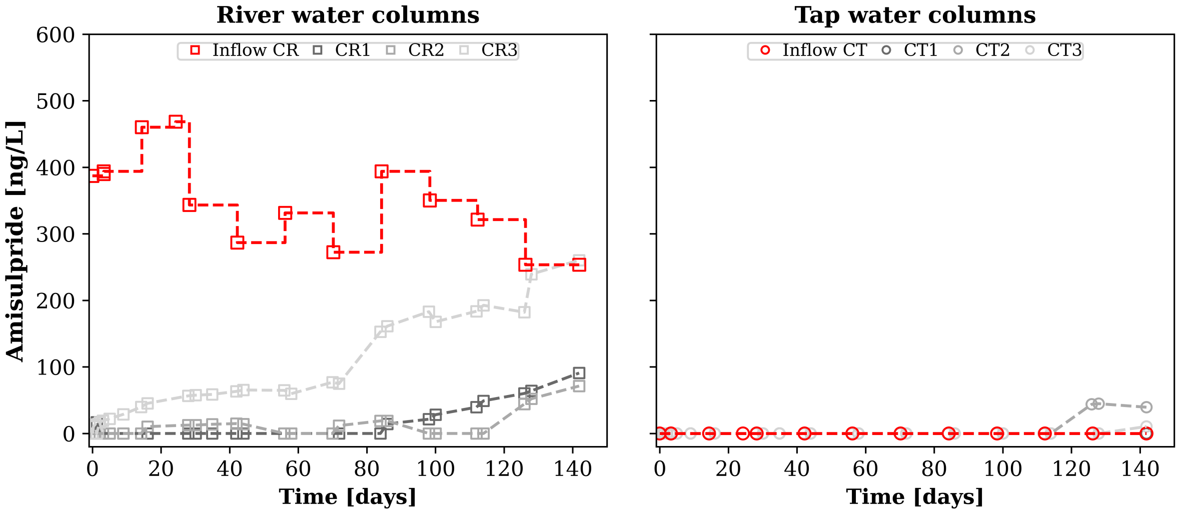

Figure S21: Evolution of Amisulpride in the six columns.

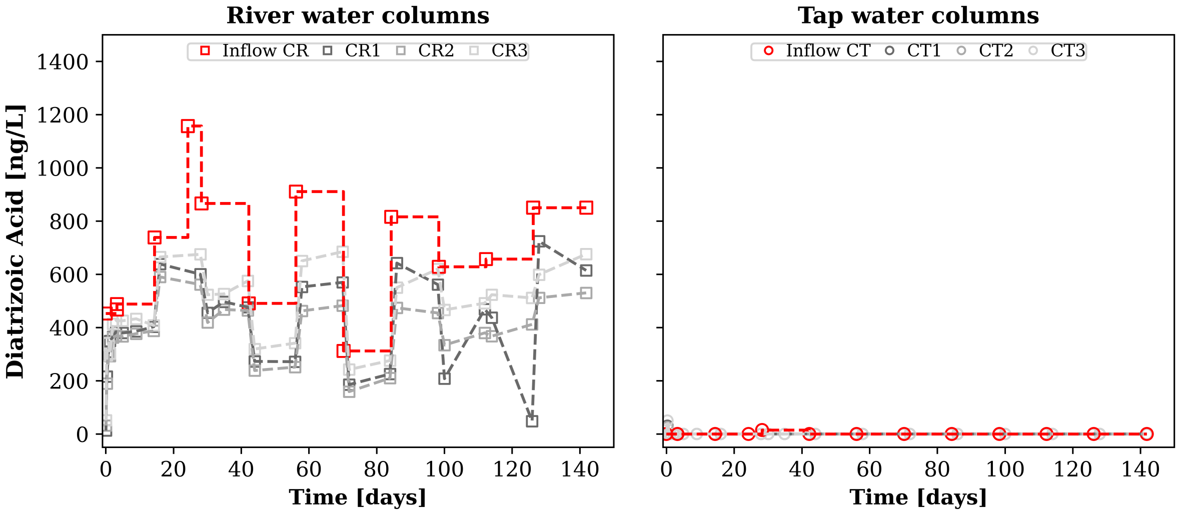

Figure S22: Evolution of Diatrizoic Acid in the six columns.

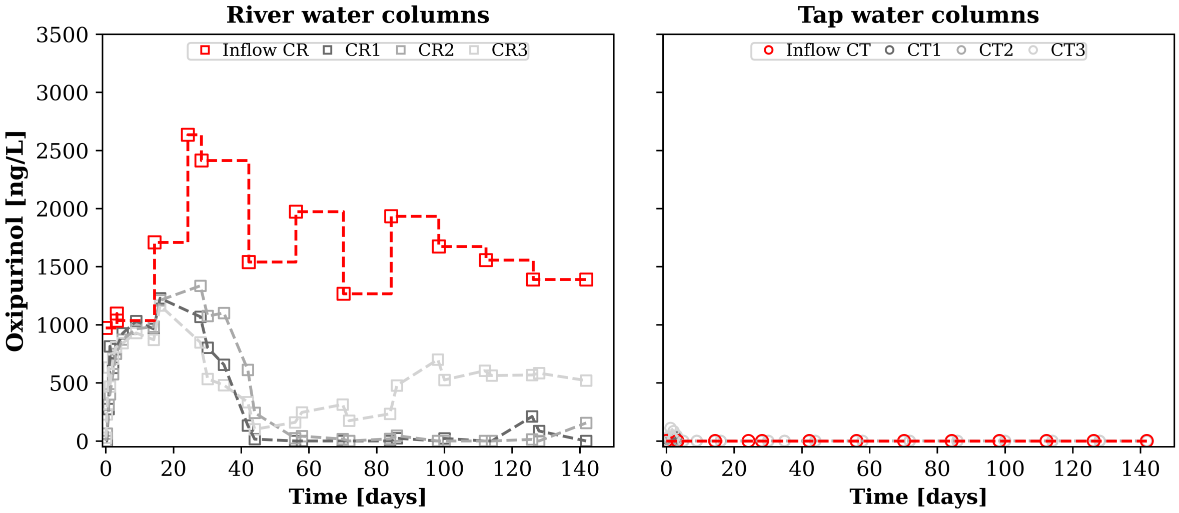

Figure S23: Evolution of Oxipurinol in the six columns.

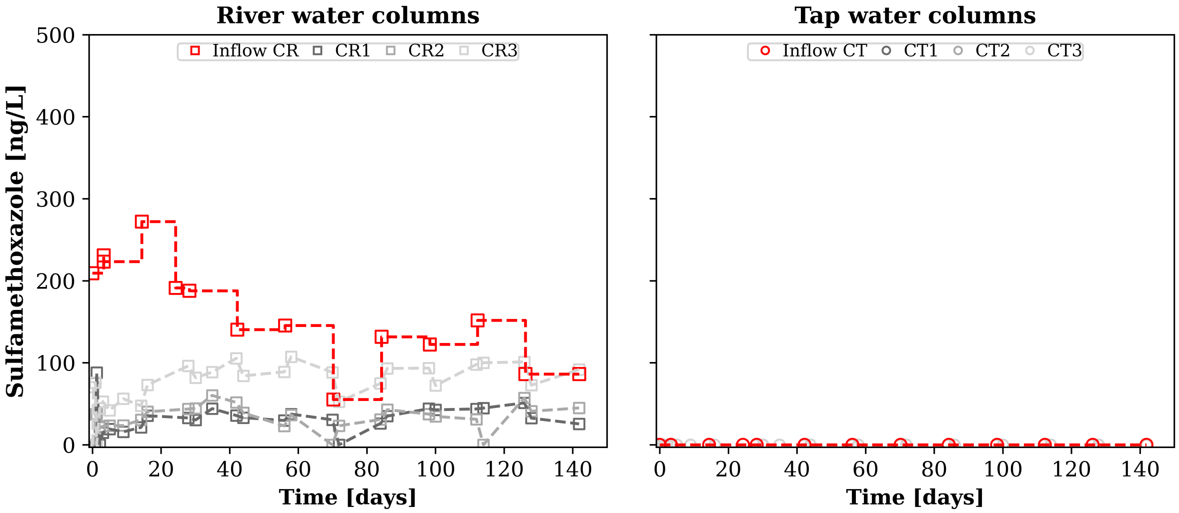

Figure S24: Evolution of Sulfamethoxazole in the six columns.

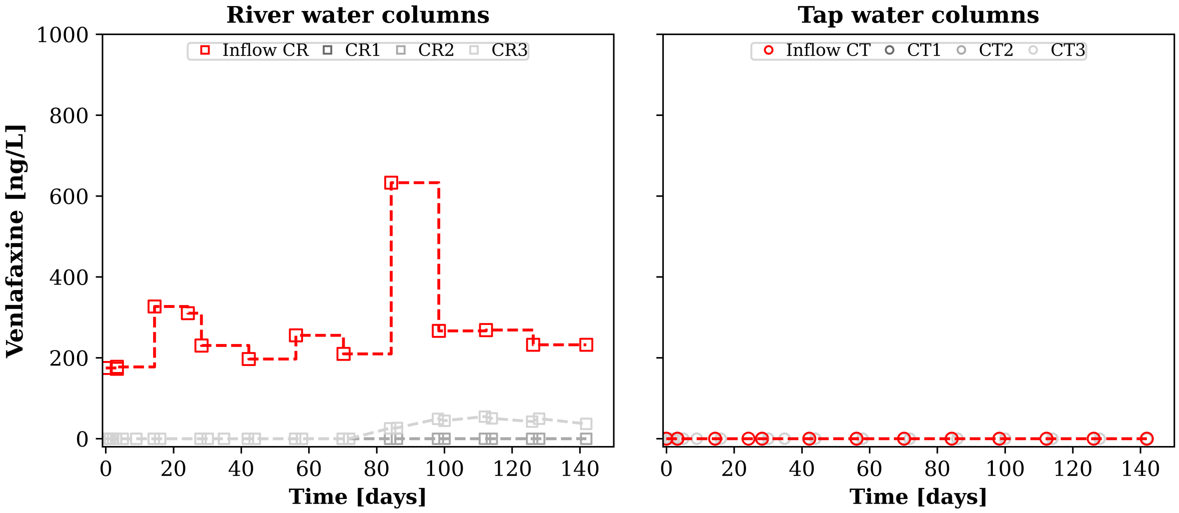

Figure S25: Evolution of Venlafaxine in the six columns.

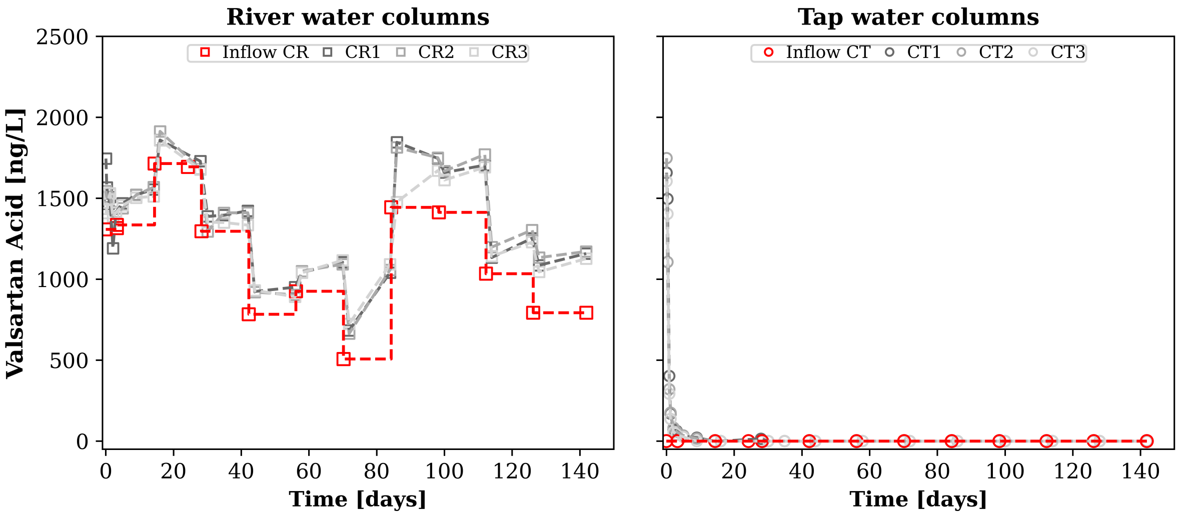

Figure S26: Evolution of Valsartan Acid in the six columns.
